# Statistical analysis of two arm randomized pre-post designs with one post-treatment measurement

**DOI:** 10.1186/s12874-021-01323-9

**Published:** 2021-07-24

**Authors:** Fei Wan

**Affiliations:** Department of Surgery, Division of Public Health Sciences, Washington University School of Medicine, Campus Box 8100, 660 S. Euclid Ave, St. Louis, MO USA

**Keywords:** Pre-post design, ANCOVA, ANOVA, Repeated measures, Change score, Treatment effect

## Abstract

**Background:**

Randomized pre-post designs, with outcomes measured at baseline and after treatment, have been commonly used to compare the clinical effectiveness of two competing treatments. There are vast, but often conflicting, amount of information in current literature about the best analytic methods for pre-post designs. It is challenging for applied researchers to make an informed choice.

**Methods:**

We discuss six methods commonly used in literature: one way analysis of variance (“***ANOVA”*)**, analysis of covariance main effect and interaction models on the post-treatment score (“***ANCOVA*I**” and “***ANCOVA*II**”), ***ANOVA*** on the change score between the baseline and post-treatment scores (“***ANOVA-Change***”), repeated measures (“***RM”***) and constrained repeated measures (“***cRM”***) models on the baseline and post-treatment scores as joint outcomes. We review a number of study endpoints in randomized pre-post designs and identify the mean difference in the post-treatment score as the common treatment effect that all six methods target. We delineate the underlying differences and connections between these competing methods in homogeneous and heterogeneous study populations.

**Results:**

***ANCOVA*** and ***cRM*** outperform other alternative methods because their treatment effect estimators have the smallest variances. ***cRM*** has comparable performance to ***ANCOVA*I** in the homogeneous scenario and to ***ANCOVA*II** in the heterogeneous scenario. In spite of that, ***ANCOVA*** has several advantages over **cRM:** i) the baseline score is adjusted as covariate because it is not an outcome by definition; ii) it is very convenient to incorporate other baseline variables and easy to handle complex heteroscedasticity patterns in a linear regression framework.

**Conclusions:**

***ANCOVA*** is a simple and the most efficient approach for analyzing pre-post randomized designs.

**Supplementary Information:**

The online version contains supplementary material available at 10.1186/s12874-021-01323-9.

## Background

Two arm parallel randomized trials have been widely used to compare the clinical effectiveness of competing treatments in improving patients’ health outcomes. In these trials, continuous outcomes of interest were routinely measured at baseline (defined as “baseline score”) and one post treatment time point (defined as “post-treatment score”). The primary purpose of designing a pre-post randomized study is to answer the scientific question of interest: is treatment ***A*** more effective than treatment ***B***? To assess the difference in the treatment effectiveness between two treatments, we need to select a study endpoint and quantify a treatment effect. Common study endpoints include the post treatment score, the change score from baseline to post treatment, a percentage change from baseline, and rate of change from baseline. The difference between two arms on selected study endpoints is defined as the treatment effect. Few studies have investigated the links between these different metrics of treatment effect in a randomized pre-post trial. These underlying connections are critical in understanding the equivalence among some statistical methods that may appear to be very different at the first sight. We need to be certain about the type of treatment effect each method targets and select the one that yields an unbiased and the most efficient estimator of the treatment effect of our interest.

There are a number of statistical methods commonly used in analyzing pre-post trials. We can analyze the post-treatment score using one way analysis of variance model (***ANOVA***) [1, 2], analysis of covariance model adjusting for the baseline score (***ANCOVA*I**) [2–7], and ANCOVA including a baseline score by treatment interaction (***ANCOVA*II**) [3, 4, 8–10]. We can also analyze the change score using ***ANOVA*** (***ANOVA-Change***) [11]. Alternatively, we can model the baseline and post-treatment scores jointly using repeated measures models (***RM***) and constrained repeated measures models (***cRM***) [10, 12–14]. Despite of the simplicity and wide application of randomized pre-post designs, which method is the best analytic approach has been a debated topic and many methodological studies have been performed to compare different statistical methods for past decades [1–13]. However, it is challenging for applied researchers to evaluate this vast, but often conflicting, amount of information in current literature and make an informed choice.

In this study we aim to review ***ANOVA, ANCOVA*I*, ANCOVA*II*, ANOVA-Change, RM,*and*cRM*** from a practical standpoint, with the focus on delineating the differences and underlying connections between them. In section Methods, we first provide notations and assumptions for a typical pre-post design, define homogeneous and heterogeneous study populations, and discuss some common study endpoints and the associated metrics of treatment effects. We next analytically assess differences and connections between these competing models in the homogeneous and heterogeneous scenarios by first describing each model using the same set of population mean, variance, and covariance parameters. In section Results, we compare the relative efficiency of these competing methods theoretically using three simulated weight loss trial examples (homogeneous data, heterogeneous data with balanced design, heterogeneous data with unbalanced design). In the last two sections, we discuss the results and give recommendation on the best analytical approach in a randomized pre-post design.

## Methods

### A hypothetical weight loss trial and metrics of treatment effects

#### Notations

In a hypothetical two arm parallel weight loss trial comparing the effect of a new drug (“treatment”) and a placebo (“control”) in reducing participants’ body weights, we use *Y*_*ijt*_ to denote body weight of the *i* th subject (*i* = 1, 2, 3, …*n*_*j*_) in the *j*th treatment arm (*j* = 0, 1) at the *t* th time (*t* = *t*_0_, *t*_1_ ). *n*_0_ and *n*_1_ are the number of subjects in the control and treatment arms.

**Fig. 1:**
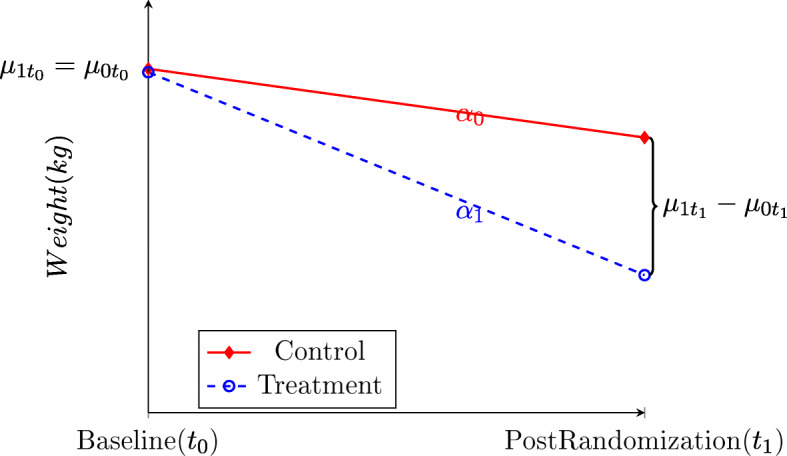
Hypothetical two arm pre-post weight loss randomized trial

We denote the mean baseline weights for the treatment and control arms by ${\mu}_{1{t}_0}$ and ${\mu}_{0{t}_0}$, respectively. Random allocation guarantees ${\mu}_{1{t}_0}={\mu}_{0{t}_0}$ and we let ${\mu}_{t_0}$ denote the overall mean baseline weight. The mean weights of the treatment and control arms at time *t*_1_ are denoted by ${\mu}_{1{t}_1}$ and ${\mu}_{0{t}_1}$, respectively (Fig. 1). We define homogeneous and heterogeneous study populations as follows:

i) ***The homogeneous scenario:*** every participant has the same pattern of variance and covariance structure for their baseline and post-treatment weights, which is parameterized as below:
  $$ \sum =\left[\begin{array}{c}{\sigma}_0^2\\ {}\rho {\sigma}_0{\sigma}_1\end{array}\right.\left.\begin{array}{c}\rho {\sigma}_0{\sigma}_1\\ {}{\sigma}_1^2\end{array}\right], $$

where ${\sigma}_0^2$ and ${\sigma}_1^2$ are the variances of the baseline and post-treatment weights, *ρ* is the correlation coefficient between the baseline and post-treatment weights.

ii) ***The heterogeneous scenario:*** variance and covariance structures of the baseline and post-treatment weights differ between the treatment and control arms. Formally, we have
  $$ {\sum}_0=\left[\begin{array}{c}{\sigma}_0^2\\ {}{\rho}_0{\sigma}_0{\sigma}_{01}\end{array}\right.\left.\begin{array}{c}{\rho}_0{\sigma}_0{\sigma}_{01}\\ {}{\sigma}_{01}^2\end{array}\right], $$

and

$$ {\sum}_1=\left[\begin{array}{c}{\sigma}_0^2\\ {}{\rho}_1{\sigma}_0{\sigma}_{11}\end{array}\right.\left.\begin{array}{c}{\rho}_1{\sigma}_0{\sigma}_{11}\\ {}{\sigma}_{11}^2\end{array}\right], $$

where ${\sigma}_0^2$ is the common variance of the baseline body weight in the control and treatment arms. Randomization guarantees that the variances of the baseline weights in both arms are equal to ${\sigma}_0^2$. ${\sigma}_{01}^2$ and ${\sigma}_{11}^2$ are the variances of the post-treatment weight in the control and treatment arms. *ρ*_0_ and *ρ*_1_ are the correlation coefficients between the baseline and post-treatment weights in the control and treatment arms, respectively. In practice, participants may respond to the treatment more differently so that variability of the post-treatment weight tends to be larger in the treatment arm than in the control arm and the correlation between pre- and post-treatment weights are usually stronger in the control arm than in the treatment arm. i.e., *ρ*_0_ > *ρ*_1_ and ${\sigma}_{11}^2>{\sigma}_{01}^2$.

#### Metrics of treatment effect

We discuss the following three metrics of treatment effect commonly reported in pre-post trials:

i) The primary endpoint is the post-treatment weight measured at *t*_1_. The difference in the mean post-treatment weights of two arms is defined as a treatment effect, which is parameterized as follows:
  $$ \tau ={\mu}_{1{t}_1}-{\mu}_{0{t}_1} $$

For example, if *τ* =  − 10, we can interpret the results as “at the end of the trial, the mean weight was 10 pounds lower in the treatment group than in the control group.”

ii) The primary endpoint is the change score calculated by subtracting the baseline weight from the post-treatment weight. i.e., ${\Delta }_{ij}={Y}_{ij{t}_1}-{Y}_{ij{t}_0}$. The difference in the mean change scores of two arms is a treatment effect. Formally, we have:
  $$ \overset{\sim }{\tau }=\left({\mu}_{1{t}_1}-{\mu}_{1{t}_0}\right)-\left({\mu}_{0{t}_1}-{\mu}_{0{t}_0}\right) $$

e.g. if $\overset{\sim }{\tau }=-10$, this difference is usually interpreted as “weight **reductions** were 10 pounds greater in the treatment group than in the control group”. Since randomization ensures ${\mu}_{0{t}_1}={\mu}_{0{t}_0}$, it follows directly $\overset{\sim }{\tau }=\tau$. When we code “0” for *t*_0_ and “1” for *t*_1_, the mean change score for each arm can also be interpreted as the mean change rate per unit time for each arm, represented by slopes in Fig. 1. Thus, the difference in slopes, denoted by $\overset{\sim }{\overset{\sim }{\tau }}={\alpha}_1-{\alpha}_0$, is also equivalent to *τ*. As shown in previous section, ***ANOVA*** and ***ANCOVA*** target *τ*, ***ANOVA-CHANGE*** targets $\overset{\sim }{\tau },$ and ***RM*** targets $\overset{\sim }{\overset{\sim }{\tau }}.\kern0.5em$ However, we can compare these statistical methods targeting seemingly very different types of treatment effects in a meaningful way because of the equivalence between *τ*, $\overset{\sim }{\tau },$ and $\overset{\sim }{\overset{\sim }{\tau }}$ in randomized pre-post designs.

iii) The primary endpoint is the percent change from baseline weight, denoted by ${\varphi}_{ij}=\frac{\left({Y}_{ij{t}_1}-{Y}_{ij{t}_0}\right)}{Y_{ij{t}_0}}$. The mean difference in the percent change between two arms is defined as a treatment effect and parameterized as follows:
  $$ {\tau}^{\ast }={\overline{\varphi}}_1-{\overline{\varphi}}_0, $$

where ${\overline{\varphi}}_1$ and ${\overline{\varphi}}_0$ are the mean percent changes of the treatment and control arms. Although the percent change is popular among clinical researchers, this metric has several drawbacks [1, 15, 16]: i) the percent change is a function of ratio $\frac{Y_{ij{t}_1}}{Y_{ij{t}_0}}$ . The distribution of the percent change is highly skewed. Analyzing it with normal-theory based statistical methods is not justified and non-parametric statistical methods are generally less powerful; ii) the percent change is not a symmetric measure. For example, the mean weight of adults over 20 in US is 197.8 pound for men and 170.5 pound for women. The mean difference is 27.3 pound between men and women. Men weight 16% (i.e.,100 × ((197.8–170.5)/170.5)) more than women, whereas women weight 13.8% (i.e., 100 × ((197.8–170.5)/197.8)) less than men. The differences could be different depending on which sex is used as devisor; iii) the percent change is not an additive measure. For example, if a participant’s weight increases by 10% in first 6 months and fall by 10% for the next 6 months, the 2 % changes do not cancel out. The participant’s weight at the end would be only 99% of the participant’s starting weight.

### Statistical models

In this section, we focus on six methods that estimate *τ*. We describe each statistical model using the same set of population mean, variance, and covariance parameters defined in section Methods for homogeneous and heterogeneous scenarios, separately. For each method, we present the closed-form expressions of the point estimator of treatment effect and its variance. It often goes unnoticed in practice that different statistical methods have different types of variances (i.e., conditional vs. unconditional variances) associated with their treatment effect estimators. For example, the OLS model-based variances for ANCOVA are conditional because OLS assumes the baseline weight is fixed. Generally speaking, the baseline weight is random because we rarely enroll participants into randomized trials based on predetermined values of the baseline weight. Thus, the unconditional variance and the corresponding unconditional inference is of greater interest because we want the findings derived from the current sample to be generalizable to the population of interest. We will discuss in details whether the OLS model-based conditional inference (i.e., test statistics and *p*-values from standard statistical softwares) for ANCOVA is still valid for unconditional hypothesis testing and the potential fixes that we can use to draw valid unconditional inference if the usual OLS model-based inference is biased.

#### When the study population is homogeneous

**Method 1:*ANOVA modeling post treatment measure (“ANOVA-Post”).*** We model the post-treatment body weight ${Y}_{ij{t}_1}$ using the binary treatment indicator *G*_*ij*_ (1 if in the treatment arm; 0 if in the control arm) as follows:

1 $$ {Y}_{ij{t}_1}={\beta}_0^{(1)}+{\beta}_1^{(1)}{G}_{ij}+{e}_{ij}^{(1)},i=1,2,\dots, {n}_j;j=0,1; $$

$$ {e}_{ij}^{(1)}\sim N\left(0,{\sigma}_1^2\right), $$

where $$ {\beta}_0^{(1)}={\mu}_{0{t}_1}, $$$$ {\beta}_1^{(1)}={\mu}_{1{t}_1}-{\mu}_{0{t}_1}= $$*τ*, and $$ {e}_{ij}^{(1)} $$ is independently and identically distributed (i.i.d) random error. $$ {\beta}_1^{(1)} $$ represents the treatment effect. Model (1) is homoscedastic with a constant residual variance $$ {\sigma}_1^2 $$.

We can fit an ordinary least squares (OLS) regression to estimate the coefficients and standard errors of model (1). The closed-form expressions of the OLS estimator ${\hat{\beta}}_{1, ols}^{(1)}$ and its “unconditional” variance, denoted by $\mathit{\operatorname{var}}\left({\hat{\beta}}_{1, ols}^{(1)}\right)$, are presented in Table 1. ${\hat{\beta}}_{1, ols}^{(1)}$ is estimated by the sample group mean difference in the post-treatment weight between two arms. ${\hat{\beta}}_{1, ols}^{(1)}$ is unbiased for *τ*. The OLS model-based variance of ${\hat{\beta}}_{1, ols}^{(1)}$ assuming known ${\sigma}_1^2$ is:

$$ {\mathit{\operatorname{var}}}_{ols}\left({\hat{\beta}}_{1, ols}^{(1)}\right)=\frac{\sigma_1^2}{\sum_{j=0}^1{\sum}_{i=1}^{n_j}{\left({G}_{ij}-{G}_{..}\right)}^2}, $$

where ${G}_{..}=\frac{\sum_{j=0}^1{\sum}_{i=1}^{n_j}{G}_{ij}}{n_0+{n}_1}=\frac{n_1}{n_0+{n}_1}$. ${\sigma}_1^2$ is estimated by

$$ {\hat{\sigma}}_1^2=\frac{\sum_{j=0}^1{\sum}_{i=1}^{n_j}{\left({y}_{ij{t}_1}-{\hat{y}}_{ij{t}_1}^{(1)}\right)}^2}{\left({n}_0+{n}_1-2\right)}, $$

where ${\hat{y}}_{ij{t}_1}^{(1)}={\hat{\beta}}_{0, ols}^{(1)}+{\hat{\beta}}_{1, ols}^{(1)}{G}_{ij}$ is the predicted value from model (1). We let ${\hat{\mathit{\operatorname{var}}}}_{ols}\left({\hat{\beta}}_{1, ols}^{(1)}\right)$ denote the OLS model-based variance estimator with ${\hat{\sigma}}_1^2$ substituted for ${\sigma}_1^2$, which is output by standard statistical softwares (Table 1). Since ${\sum}_{j=0}^1{\sum}_{i=1}^{n_j}{\left({G}_{ij}-{G}_{..}\right)}^2=\frac{n_0{n}_1}{n_0+{n}_1}$, it follows that ${\mathit{\operatorname{var}}}_{ols}\left({\hat{\beta}}_{1, ols}^{(1)}\right)=\mathit{\operatorname{var}}\left({\hat{\beta}}_{1, ols}^{(1)}\right)$. It is well established that ${\hat{\mathit{\operatorname{var}}}}_{ols}\left({\hat{\beta}}_{1, ols}^{(1)}\right)$ is unbiased for ${\mathit{\operatorname{var}}}_{ols}\left({\hat{\beta}}_{1, ols}^{(1)}\right)$. Thus, ${\hat{\mathit{\operatorname{var}}}}_{ols}\left({\hat{\beta}}_{1, ols}^{(1)}\right)$ is unbiased for $\mathit{\operatorname{var}}\left({\hat{\beta}}_{1, ols}^{(1)}\right)$. The usual OLS model-based inference (i.e., test statistics $t=\frac{{\hat{\beta}}_{1, ols}^{(1)}}{\sqrt{{\hat{va\mathrm{r}}}_{ols}\left({\hat{\beta}}_{1, ols}^{(1)}\right)\ }}$ and the associated *p*-value) is valid for testing *H*_*o*_ : *τ* = 0 unconditionally.

**Table 1:** Estimators of treatment effect and variance estimators in a homogeneous study population

Model	Estimator of treatment effect (*τ*)	Type^a^	True variance of treatment effect estimator	OLS model based variance estimator
*ANOVA-Post*	${\hat{\beta}}_{1, ols}^{(1)}={\overline{y}}_{.1{t}_1}-{\overline{y}}_{.0{t}_1}$	U	$\mathit{\operatorname{var}}\left({\hat{\beta}}_{1, ols}^{(1)}\right)=\frac{\sigma_1^2}{n_0}+\frac{\sigma_1^2}{n_1}$	${\hat{\mathit{\operatorname{var}}}}_{ols}\left({\hat{\beta}}_{1, ols}^{(1)}\right)=\frac{{\hat{\sigma}}_1^2}{\sum_{j=0}^1{\sum}_{i=1}^{n_j}{\left({G}_{ij}-{G}_{..}\right)}^2}$ ${\hat{\sigma}}_1^2=\frac{\sum_{j=0}^1{\sum}_{i=1}^{n_j}{\left({y}_{ij{t}_1}-{\hat{y}}_{ij{t}_1}\right)}^2}{\left({n}_0+{n}_1-2\right)}$
*ANCOVA-Post I*	${\hat{\beta}}_{1, ols}^{(2)}=\left({\overline{y}}_{.1{t}_1}-{\overline{y}}_{.0{t}_1}\right)-{\hat{\beta}}_{2, ols}^{(2)}\left({\overline{y}}_{.1{t}_0}-{\overline{y}}_{.0{t}_0}\right)$	C	$\mathit{\operatorname{var}}\left({\hat{\beta}}_{1, ols}^{(2)}|{Y}_{ij{t}_0}\right)=\left(\frac{1}{n_0}+\frac{1}{n_1}+\frac{{\left({\overline{y}}_{.1{t}_0}-{\overline{y}}_{.0{t}_0}\right)}^2}{\sum_{j=0}^1{\sum}_{i=1}^{n_j}{\left({y}_{ij{t}_0}-{\overline{y}}_{.j{t}_0}\right)}^2}\right){\sigma}_{\epsilon^{(2)}}^2$, ${\sigma}_{\epsilon^{(2)}}^2=\left(1-{\rho}^2\right){\sigma}_1^2$	${\hat{\mathit{\operatorname{var}}}}_{ols}\Big({\hat{\beta}}_{1, ols}^{(2)}\left|{Y}_{ij{t}_0}\right)=\left(\frac{1}{n_0}+\frac{1}{n_1}+\frac{{\left({\overline{y}}_{.1{t}_0}-{\overline{y}}_{.0{t}_0}\right)}^2}{\sum_{j=0}^1{\sum}_{i=1}^{n_j}{\left({y}_{ij{t}_0}-{\overline{y}}_{.j{t}_0}\right)}^2}\right){\hat{\sigma}}_{e_{ij}^{(2)}}^2$, ${\hat{\sigma}}_{e_{ij}^{(2)}}^2=\frac{\sum_{j=0}^1{\sum}_{i=1}^{n_j}{\left({y}_{ij{t}_1}-{\hat{y}}_{ij{t}_1}\right)}^2}{\left({n}_0+{n}_1-4\right)}$
		U	$\mathit{\operatorname{var}}\left({\hat{\beta}}_{1, ols}^{(2)}\right)=\left(\frac{1}{n_0}+\frac{1}{n_1}\right)\left(1-{\rho}^2\right){\sigma}_1^2$	
*RM*	${\hat{\gamma}}_{3,\kern0.5em gls}^{(3)}=\left({\overline{y}}_{.1{t}_1}-{\overline{y}}_{.1{t}_0}\right)-\left({\overline{y}}_{.0{t}_1}-{\overline{y}}_{.0{t}_0}\right)$	U	$\mathit{\operatorname{var}}\left({\hat{\gamma}}_{3,\kern0.5em gls}^{(3)}\right)=\left(\frac{1}{n_0}+\frac{1}{n_1}\right)\left({\sigma}_1^2+{\sigma}_0^2-2\rho {\sigma}_0{\sigma}_1\right)$	
*cRM*	${\hat{\gamma}}_{3,\kern0.5em gls}^{(4)}=\left({\overline{y}}_{.1{t}_1}-{\overline{y}}_{.0{t}_1}\right)-\frac{\rho {\sigma}_0{\sigma}_1}{\sigma_0^2}\left({\overline{y}}_{.1{t}_0}-{\overline{y}}_{.0{t}_0}\right)$	U	$\mathit{\operatorname{var}}\left({\hat{\gamma}}_{3, gls}^{(4)}\right)=\left(\frac{1}{n_0}+\frac{1}{n_1}\right)\left(1-{\rho}^2\right){\sigma}_1^2$	
*ANOVA-Change*	${\hat{\beta}}_{1, ols}^{(5)}=\left({\overline{y}}_{.1{t}_1}-{\overline{y}}_{.1{t}_0}\right)-\left({\overline{y}}_{.0{t}_1}-{\overline{y}}_{.0{t}_0}\right)$	U	$\mathit{\operatorname{var}}\left({\hat{\beta}}_{1, ols}^{(5)}\right)=\left(\frac{1}{n_0}+\frac{1}{n_1}\right)\left({\sigma}_1^2+{\sigma}_0^2-2\rho {\sigma}_0{\sigma}_1\right)$	${\hat{\mathit{\operatorname{var}}}}_{ols}\left({\hat{\beta}}_{1, ols}^{(5)}\right)=\frac{{\hat{\sigma}}_{\epsilon^{(5)}}^2}{\sum_{j=0}^1{\sum}_{i=1}^{n_j}{\left({G}_{ij}-{G}_{..}\right)}^2},$ ${\hat{\sigma}}_{\epsilon^{(5)}}^2=\frac{\sum_{j=0}^1{\sum}_{i=1}^{n_j}{\left({\Delta }_{ij}-{\hat{\Delta }}_{ij}^{(5)}\right)}^2}{\left({n}_0+{n}_1-2\right)}$

^a^U- unconditional variance; C- conditional variance

**Method 2:*ANCOVA modeling post treatment measure (“ANCOVA*I*”)*:** We model the post-treatment weight ${Y}_{ij{t}_1}$ using the binary treatment indicator *G*_*ij*_ and the baseline weight ${Y}_{ij{t}_0}$.

2 $$ {Y}_{ij{t}_1}={\beta}_0^{(2)}+{\beta}_1^{(2)}{G}_{ij}+{\beta}_2^{(2)}{Y}_{ij{t}_0}+{e}_{ij}^{(2)},i=1,2,\dots, {n}_j;j=0,1; $$

$$ {e}_{ij}^{(2)}\sim N\left(0,{\sigma}_{\epsilon^{(2)}}^2\right)\ \mathrm{and}\ {\sigma}_{\epsilon^{(2)}}^2=\left(1-{\rho}^2\right){\sigma}_1^{2.} $$

, where ${\beta}_0^{(2)}={\mu}_{0{t}_1}-\rho \frac{\sigma_1}{\sigma_0}{\mu}_{t_0}$, ${\beta}_1^{(2)}$ = $\tau, \kern0.5em {\beta}_2^{(2)}$ = $\rho \frac{\sigma_1}{\sigma_0}$, and ${e}_{ij}^{(2)}$ is i.i.d random error. ${\beta}_1^{(2)}$ measures the treatment effect *τ* and ${\beta}_2^{(2)}$ represents the slope of the pre-post association between ${Y}_{ij{t}_1}$ and ${Y}_{ij{t}_0}$. Model (2) has a common residual variance ${\sigma}_{\epsilon^{(2)}}^2$ and implicitly assumes that two arms share the common baseline mean ${\mu}_{t_0}$.

The coefficients and standard errors of model (2) are also estimated using an OLS regression. The OLS estimator ${\hat{\beta}}_{1, ols}^{(2)}$ is derived as the sample mean difference in the post-treatment weight adjusting for the sample mean difference in the baseline weight between two arms. The group mean difference in the baseline weight can be seen as chance imbalance in a randomized trial. ${\hat{\beta}}_{1, ols}^{(2)}$ is unbiased for *τ* both conditional on ${Y}_{ij{t}_0}$ and unconditionally. The formulas of ${\hat{\beta}}_{1, ols}^{(2)}$ and its “unconditional” variance $\mathit{\operatorname{var}}\left({\hat{\beta}}_{1, ols}^{(2)}\right)$ are listed in Table 1. However, OLS assumes that the baseline weight ${Y}_{ij{t}_0}$ is fixed. OLS targets the conditional variance of ${\hat{\beta}}_{1, ols}^{(2)}$, denoted by $\mathit{\operatorname{var}}\Big({\hat{\beta}}_{1, ols}^{(2)}\left|{Y}_{ij{t}_0}\right)$, instead of $\mathit{\operatorname{var}}\left({\hat{\beta}}_{1, ols}^{(2)}\right)$. The formula of $\mathit{\operatorname{var}}\Big({\hat{\beta}}_{1, ols}^{(2)}\left|{Y}_{ij{t}_0}\right)$ with a known common residual variance ${\sigma}_{\epsilon^{(2)}}^2$ is presented in Table 1. Since ${\sigma}_{\epsilon^{(2)}}^2$ is generally unknown, it is estimated by the following sample residual variance:

$$ {\hat{\sigma}}_{e_{ij}^{(2)}}^2=\frac{\sum_{j=0}^1{\sum}_{i=1}^{n_j}{\left({y}_{ij{t}_1}-{\hat{y}}_{ij{t}_1}^{(2)}\right)}^2}{\left({n}_0+{n}_1-3\right)} $$

, where ${\hat{y}}_{ij{t}_1}^{(2)}={\hat{\beta}}_{0, ols}^{(2)}+{\hat{\beta}}_{1, ols}^{(2)}{G}_{ij}+{\hat{\beta}}_{2, ols}^{(2)}{Y}_{ij{t}_0}$, the predicted value from model (2). We let ${\hat{\mathit{\operatorname{var}}}}_{ols}\left({\hat{\beta}}_{1, ols}^{(2)}|{Y}_{ij{t}_0}\right)$ denote the OLS model-based variance estimator with ${\hat{\sigma}}_{\epsilon^{(2)}}^2$ substituted for ${\sigma}_{\epsilon^{(2)}}^2$ . Note that ${\hat{\mathit{\operatorname{var}}}}_{ols}\left({\hat{\beta}}_{1, ols}^{(2)}|{Y}_{ij{t}_0}\right)$ is reported by standard statistical softwares (e.g. “proc reg” in SAS). Its formula is presented in Table 1.

Since we want to generalize our conclusions to a general population and ${Y}_{ij{t}_0}$ can take different values from those collected in the current sample, we may wonder whether significance tests based on the model-based conditional variance assuming ${Y}_{ij{t}_0}$ is fixed (e.g., $t=\frac{{\hat{\beta}}_{1, ols}^{(2)}}{\sqrt{{\hat{\mathit{\operatorname{var}}}}_{ols}\left({\hat{\beta}}_{1, ols}^{(2)}|{Y}_{ij{t}_0}\right)\kern0.5em }}$) is comparable to unconditional inference (e.g., $t=\frac{{\hat{\beta}}_{1, ols}^{(2)}}{\sqrt{\mathit{\operatorname{var}}\left({\hat{\beta}}_{1, ols}^{(2)}\right)}}$), in which ${Y}_{ij{t}_0}$ is treated as random variable, for testing *H*_*o*_ : *τ* = 0. To establish this equivalence, we need to show: i) ${\hat{\mathit{\operatorname{var}}}}_{ols}\left({\hat{\beta}}_{1, ols}^{(2)}|{Y}_{ij{t}_0}\right)$ is unbiased for $\mathit{\operatorname{var}}\Big({\hat{\beta}}_{1, ols}^{(2)}\left|{Y}_{ij{t}_0}\right)$; ii) $\mathit{\operatorname{var}}\left({\hat{\beta}}_{1, ols}^{(2)}|{Y}_{ij{t}_0}\right)$ is unbiased for $\mathit{\operatorname{var}}\left({\hat{\beta}}_{1, ols}^{(2)}\right)$. The first part is well established in a homoscedastic linear model. The second part holds because we can show that $\kern0.50em \mathit{\operatorname{var}}\left({\hat{\beta}}_{1, ols}^{(2)}\right)$ =E($\mathit{\operatorname{var}}\left({\hat{\beta}}_{1, ols}^{(2)}|{Y}_{ij{t}_0}\right)$) using the law of total variance formula and the fact that ${\hat{\beta}}_{1, ols}^{(2)}$ is unbiased for *τ*. That is, the unconditional variance of ${\hat{\beta}}_{1, ols}^{(2)}$ is the average of its conditional variance over the distribution of the baseline weight. Therefore, the usual model-based standard errors and associated *p*-values are valid for unconditional inference [3, 5, 17].

**Method 3:*Repeated measures model (“RM”)*:*RM*** models the baseline and post-treatment weights (${Y}_{ij{t}_0}$, ${Y}_{ij{t}_1}$) jointly using the binary treatment indicator *G*_*ij*_, the binary time factor *T*_*ij*_, the time by treatment interaction *G*_*ij*_ × *T*_*ij*_ as follows:

3 $$ {Y}_{ij t}={\gamma}_0^{(3)}+{\gamma}_1^{(3)}{G}_{ij}+{\gamma}_2^{(3)}{T}_{ij}+{\gamma}_3^{(3)}{G}_{ij}\times {T}_{ij}+{e}_{ij t}^{(3)},i=1,2,\dots, {n}_j;j=0,1;t={t}_0,{t}_1, $$

$$ \left(\begin{array}{c}{e}_{ij{t}_0}^{(3)}\\ {}{e}_{ij{t}_1}^{(3)}\end{array}\right)\sim N\left(\left[\begin{array}{c}0\\ {}0\end{array}\right],\sum \right), $$

When *t*_0_ = 0 and *t*_1_ = 1, $$ \kern0.50em {\gamma}_0^{(3)}= $$$$ {\mu}_{0{t}_0} $$, $$ {\gamma}_1^{(3)}={\mu}_{1{t}_0}-{\mu}_{0{t}_0}, $$$$ {\gamma}_2^{(3)}={\mu}_{0{t}_1}-{\mu}_{0{t}_0}, $$ and $$ {\gamma}_3^{(3)}=\left({\mu}_{1{t}_1}-{\mu}_{1{t}_0}\right)-\left({\mu}_{0{t}_1}-{\mu}_{0{t}_0}\right) $$. $$ {\gamma}_0^{(3)} $$ represents the mean baseline weight of the control arm, $$ {\gamma}_1^{(3)} $$ represents the difference in the mean baseline weights of the treatment and control arms, $$ {\gamma}_2^{(3)} $$ represents the mean change from baseline in the control arm, and $$ {\gamma}_3^{(3)} $$ is generally interpreted as the difference in the mean change from baseline in a unit time interval between the treatment and control arms (“difference in difference”), also known as the difference in slopes. We have $$ {\mu}_{1{t}_0}={\mu}_{0{t}_0} $$ from random allocation and it follows that $$ {\gamma}_1^{(3)}=0 $$ and $$ {\gamma}_3^{(3)}={\mu}_{1{t}_1}-{\mu}_{1{t}_1}=\tau . $$ Thus, testing $$ {H}_o:{\gamma}_3^{(3)}=0 $$ is equivalent to testing *H*_*o*_ : *τ* = 0.

The generalized least squares (GLS) model with correlated outcomes is routinely used to estimate the coefficients and standard errors of model (3). The GLS estimator of the treatment effect ${\hat{\gamma}}_{3,\kern0.5em gls}^{(3)}$ and its variance $\mathit{\operatorname{var}}\Big({\hat{\gamma}}_{3,\kern0.5em gls}^{(3)}$) given known variance and covariance parameters are presented in Table 1. ${\hat{\gamma}}_{3,\kern0.5em gls}^{(3)}$ is estimated by the sample mean difference in body weight change between two arms and is unbiased for τ in a large sample. The variance and covariance parameters are generally unknown and need to be estimated using the restricted maximum likelihood (REML). The conventional maximal likelihood estimation (MLE) should be avoided. The REML variance estimator ${\hat{\ \mathit{\operatorname{var}}}}_{reml}\Big({\hat{\gamma}}_{3,\kern0.5em gls}^{(3)}$) is derived by plugging the REML estimators of the variance and covariance parameters (i.e., ${\sigma}_0^2,{\sigma}_1^2,\rho {\sigma}_0{\sigma}_1$) into the formula of $\mathit{\operatorname{var}}\left({\hat{\gamma}}_{3,\kern0.5em gls}^{(3)}\right)$.We use Kenward and Roger method [18](“ddfm = kenwardroger” in SAS proc. mixed procedure) to adjust for the potential finite sample bias in ${\hat{\ \mathit{\operatorname{var}}}}_{reml}\Big({\hat{\gamma}}_{3,\kern0.5em gls}^{(3)}$) because of its failure to incorporate variabilities of the REML estimators of the variance and covariance parameters. This adjustment involves inflating the variance and covariance matrix and computing an adjusted approximation degrees of freedom.

**Method 4:*Constrained Repeated measures Model (“cRM”)*:** By specifying ${\gamma}_1^{(3)}$ in the model, ***RM*** model (3) assumes the mean baseline weight is different between two arms. Liang and Zeger [8] proposed the following ***cRM*** model by fixing ${\gamma}_1^{(3)}=0$ to force the treatment and control arms to have the same intercept. Intuitively, ***cRM*** is more efficient than ***RM*** because ***cRM*** estimates one less parameter. Formally, we model the baseline and post-treatment weights (${Y}_{ij{t}_0}$, ${Y}_{ij{t}_1}$) jointly using the binary factor *T*_*ij*_, a time by treatment interaction *G*_*ij*_ × *T*_*ij*_ in the following ***cRM*** model:

4 $$ {Y}_{ij t}={\gamma}_0^{(4)}+{\gamma}_2^{(4)}{T}_{ij}+{\gamma}_3^{(4)}{G}_{ij}\times {T}_{ij}+{e}_{ij t}^{(4)},i=1,2,\dots, {n}_j;j=0,1;t={t}_0,{t}_1 $$

$$ \left(\begin{array}{c}{e}_{ij{t}_0}^{(4)}\\ {}{e}_{ij{t}_1}^{(4)}\end{array}\right)\sim N\left(\left[\begin{array}{c}0\\ {}0\end{array}\right],\sum \right), $$

where ${\gamma}_0^{(4)}={\mu}_{t_0},{\gamma}_2^{(4)}={\mu}_{0{t}_1}-{\mu}_{0{t}_0}$, and ${\gamma}_3^{(4)}=\tau$. Interpretations of ${\gamma}_0^{(4)}$, ${\gamma}_2^{(4)}$, and ${\gamma}_3^{(4)}$ are the same as their counterparts in ***RM***. The formulas of the GLS point estimator ${\hat{\gamma}}_{3,\kern0.5em gls}^{(4)}$ and its variance $\mathit{\operatorname{var}}\left({\hat{\gamma}}_{3,\kern0.5em gls}^{(4)}\right)$ are listed in Table 1. ${\hat{\gamma}}_{3,\kern0.5em gls}^{(4)}$ is unbiased for *τ* asymptotically. The empirical or the model-based variance estimate for $\mathit{\operatorname{var}}\left({\hat{\gamma}}_{3,\kern0.5em gls}^{(4)}\right)$ is derived using REML in the same way as a regular ***RM*** model.

**Method 5:*ANOVA with change score (“ANOVA-Change”)*:** We model change score ${\Delta }_{ij}={Y}_{ij{t}_1}-{Y}_{ij{t}_0}$ using the binary treatment indicator *G*_*ij*_ as follows:

5 $$ {\Delta }_{ij}={\beta}_0^{(5)}+{\beta}_1^{(5)}{G}_{ij}+{e}_{ij}^{(5)},i=1,2,\dots, {n}_j;j=0,1; $$

$$ {e}_{ij}^{(5)}\sim N\left(0,{\sigma}_{\epsilon^{(5)}}^2\right)\ \mathrm{and}\ {\sigma}_{\epsilon^{(5)}}^2={\sigma}_1^2+{\sigma}_0^2-2\rho {\sigma}_0{\sigma}_1, $$

where ${\beta}_0^{(5)}={\mu}_{0{t}_1}-{\mu}_{0{t}_0}$, ${\beta}_1^{(5)}=\left({\mu}_{1{t}_1}-{\mu}_{1{t}_0}\right)-\left({\mu}_{0{t}_1}-{\mu}_{0{t}_0}\right)$, and ${e}_{ij}^{(3)}$ is i.i.d random error. ${\beta}_0^{(5)}$ measures the mean difference score in the control arm. ${\beta}_1^{(5)}$ measures the treatment effect $\overset{\sim }{\tau }$. Since ${\mu}_{1{t}_0}={\mu}_{0{t}_0}$ due to randomization at baseline, ${\beta}_1^{(5)}$ is reduced to *τ*. The closed-form expressions of ${\hat{\beta}}_{1, ols}^{(5)}$ and $\mathit{\operatorname{var}}\left({\hat{\beta}}_{1, ols}^{(5)}\right)$ are listed in Table 1. ${\hat{\beta}}_{1, ols}^{(5)}$ is derived as the sample mean difference in the change score between two arms (“difference in difference”) and is unbiased for *τ*. The OLS model-based variance of ${\hat{\beta}}_{1, ols}^{(5)}$ assuming known ${\sigma}_{\epsilon^{(5)}}^2$ is

$$ {\mathit{\operatorname{var}}}_{ols}\left({\hat{\beta}}_{1, ols}^{(5)}\right)=\frac{\sigma_{\epsilon^{(5)}}^2}{\sum_{j=0}^1{\sum}_{i=1}^{n_j}{\left({G}_{ij}-{G}_{..}\right)}^2}, $$

where ${G}_{..}=\frac{\sum_{j=0}^1{\sum}_{i=1}^{n_j}{G}_{ij}}{n_0+{n}_1}=\frac{n_1}{n_0+{n}_1}$. ${\sigma}_{\epsilon^{(5)}}^2$ is estimated by

$$ {\hat{\sigma}}_{\epsilon^{(5)}}^2=\frac{\sum_{j=0}^1{\sum}_{i=1}^{n_j}{\left({\Delta }_{ij}-{\hat{\Delta }}_{ij}^{(5)}\right)}^2}{\left({n}_0+{n}_1-2\right)}, $$

where ${\hat{\Delta }}_{ij}^{(5)}$ is the fitted value from model (5). We let ${\hat{\mathit{\operatorname{var}}}}_{ols}\left({\hat{\beta}}_{1, ols}^{(5)}\right)$ denote the OLS model-based variance estimator with ${\hat{\sigma}}_{\epsilon^{(5)}}^2$ substituted for ${\sigma}_{\epsilon^{(5)}}^2$ Table 1, which is reported by standard statistical softwares. Since ${\sum}_{j=0}^1{\sum}_{i=1}^{n_j}{\left({G}_{ij}-{G}_{..}\right)}^2=\frac{n_0{n}_1}{n_0+{n}_1}$, it follows that ${\mathit{\operatorname{var}}}_{ols}\left({\hat{\beta}}_{1, ols}^{(5)}\right)=\mathit{\operatorname{var}}\left({\hat{\beta}}_{1, ols}^{(5)}\right)$. It is well established that ${\hat{\mathit{\operatorname{var}}}}_{ols}\left({\hat{\beta}}_{1, ols}^{(5)}\right)$ is unbiased for ${\mathit{\operatorname{var}}}_{ols}\left({\hat{\beta}}_{1, ols}^{(5)}\right)$, and thus for $\mathit{\operatorname{var}}\left({\hat{\beta}}_{1, ols}^{(5)}\right)$. The usual OLS model-based inference is valid for unconditional hypothesis testing.

#### When the study population is heterogeneous

**Method 6:*ANCOVA*II:** Different variance and covariance structures in the treatment and control arms suggest a baseline measurement by treatment interaction term in ANCOVA [2, 3, 9, 10]. To estimate *τ* using an interaction model, we first compute the mean centered baseline weight ${\overset{\sim }{Y}}_{ij{t}_0}$ by subtracting the overall mean baseline weight from individual baseline weights. i.e., ${\overset{\sim }{Y}}_{ij{t}_0}={Y}_{ij{t}_0}-{\mu}_{t_0}$. We then model the post-treatment body weight ${Y}_{ij{t}_1}$ using the binary treatment indicator *G*_*ij*_, the mean centered baseline weight ${\overset{\sim }{Y}}_{ij{t}_0}$, and the baseline weight by treatment interaction ${G}_{ij}\times {\overset{\sim }{Y}}_{ij{t}_0}$ as follows:

6 $$ {Y}_{ij{t}_1}={\beta}_0^{(6)}+{\beta}_1^{(6)}{G}_{ij}+{\beta}_2^{(6)}{\overset{\sim }{Y}}_{ij{t}_0}+{\beta}_3^{(6)}{G}_{ij}\times {\overset{\sim }{Y}}_{ij{t}_0}+{e}_{ij}^{(6)},i=1,2,\dots, {n}_j;j=0,1; $$

$$ {e}_{i0}^{(6)}\sim N\left(0,\kern0.5em {\sigma}_{\epsilon_0^{(6)}}^2\right)\kern0.50em \mathrm{and}\ {\sigma}_{\epsilon_0^{(6)}}^2=\left(1-{\rho}_0^2\right){\sigma}_{01}^2 $$

$$ {e}_{i1}^{(6)}\sim N\left(0,\kern0.5em {\sigma}_{\epsilon_1^{(6)}}^2\right)\ \mathrm{and}\ {\sigma}_{\epsilon_1^{(6)}}^2=\left(1-{\rho}_1^2\right){\sigma}_{11}^2 $$

, where $$ {\beta}_0^{(6)}={\mu}_{0{t}_1} $$, $$ {\beta}_1^{(6)}=\tau, $$$$ {\beta}_2^{(6)}={\rho}_0\frac{\sigma_{0{t}_0}}{\sigma_0} $$, and $$ {\beta}_3^{(6)}= $$$$ {\rho}_1\frac{\sigma_{1{t}_1}}{\sigma_0}-{\rho}_0\frac{\sigma_{0{t}_0}}{\sigma_0} $$. $$ {e}_{i0}^{(6)} $$ and $$ {e}_{i1}^{(6)} $$ are i.i.d random errors in the control and treatment arms. $$ {\beta}_1^{(6)} $$ measures the treatment effect. $$ {\beta}_2^{(6)} $$ is the regression slope of the baseline body weight in the control arm. $$ {\beta}_3^{(6)} $$ measures the difference in the regression slopes of the baseline weight between the treatment and control arms. Model (6) is heteroscedastic because the error terms in the treatment and control arms have different residual variances.

As presented in Table 2, the OLS estimator ${\hat{\beta}}_{1, ols}^{(6)}$ is the adjusted mean difference in the post-treatment body weights controlling for a weighted mean difference of the baseline body weights between two arms with unequal weighting coefficients for treatment and control arms (i.e., ${\hat{\beta}}_{2, ols}^{(6)}+{\hat{\beta}}_{3, ols}^{(6)}$ for the treatment group, and ${\hat{\beta}}_{2, ols}^{(6)}$ for the control group). ${\hat{\beta}}_{1, ols}^{(6)}$ is unbiased for *τ*. The conditional variance of ${\hat{\beta}}_{1, ols}^{(6)}$, denoted by $\mathit{\operatorname{var}}\left({\hat{\beta}}_{1, ols}^{(6)}|{\overset{\sim }{Y}}_{ij{t}_0}\right)$, incorporates two different residual variances ${\sigma}_{\epsilon_0^{(6)}}^2$ and $\kern0.5em {\sigma}_{\epsilon_1^{(6)}}^2$ (Table 2). Standard statistical softwares such as SAS do not output $\mathit{\operatorname{var}}\left({\hat{\beta}}_{1, ols}^{(6)}|{\overset{\sim }{Y}}_{ij{t}_0}\right)$ because OLS incorrectly assumes a common residual variance ${\sigma}_{\epsilon^{(6)}}^2$, which is the following weighted average of $\kern0.5em {\sigma}_{\epsilon_0^{(6)}}^2$ and $\kern0.5em {\sigma}_{\epsilon_1^{(6)}}^2$:

$$ {\sigma}_{\epsilon^{(6)}}^2=\frac{n_0}{n_0+{n}_1}\kern0.5em {\sigma}_{\epsilon_0^{(6)}}^2+\frac{n_1}{n_0+{n}_1}{\sigma}_{\epsilon_1^{(6)}}^2 $$

**Table 2:** Estimators of treatment effect and variance estimators in a heterogeneous study population

Model	Estimator of treatment effect (τ)	Type	True variance of treatment effect estimator	Variance estimator from OLS model
*ANCOVA-Post* II	${\hat{\beta}}_{1, ols}^{(6)}=\left({\overline{y}}_{.1{t}_1}-\left({\hat{\beta}}_{2, ols}^{(6)}+{\hat{\beta}}_{3, ols}^{(6)}\right){\overline{\overset{\sim }{y}}}_{.1{t}_0}\right)-\left({\overline{y}}_{.0{t}_0}-{\hat{\beta}}_{2, ols}^{(6)}{\overline{\overset{\sim }{y}}}_{.0{t}_0}\right)$	C	$\mathit{\operatorname{var}}\left({\hat{\beta}}_{1, ols}^{(6)}|{\overset{\sim }{Y}}_{ij{t}_0}\right)=\left(\frac{1}{n_0}+\frac{{\overline{\overset{\sim }{y}}}_{.o{t}_0}^2}{\sum_{i=1}^{n_0}{\left(\tilde{y}_{{i}0{t}_0}-{\overline{\overset{\sim }{y}}}_{.0{t}_0}\right)}^2}\right)\ {\sigma}_{\epsilon_0^{(6)}}^2+\left(\frac{1}{n_1}+\frac{{\overline{\overset{\sim }{y}}}_{.1{t}_0}^2}{\sum_{i=1}^{n_0}{\left(\tilde{y}_{{i}1{t}_0}-{\overline{\overset{\sim }{y}}}_{.1{t}_0}\right)}^2}\right)\ {\sigma}_{\epsilon_1^{(6)}}^2$ $\kern0.5em {\sigma}_{\epsilon_0^{(6)}}^2=\left(1-{\rho}_0^2\right){\sigma}_{01}^2$, $\kern0.5em {\sigma}_{\epsilon_1^{(6)}}^2=\left(1-{\rho}_1^2\right){\sigma}_{11}^2$	${\hat{\mathit{\operatorname{var}}}}_{ols}\left({\hat{\beta}}_{1, ols}^{(6)}|{\overset{\sim }{Y}}_{ij{t}_0}\right)=\left(\frac{1}{n_0}+\frac{1}{n_1}+\frac{{\overline{\overset{\sim }{y}}}_{.o{t}_0}^2}{\sum_{i=1}^{n_0}{\left(\tilde{y}_{{i}0{t}_0}-{\overline{\overset{\sim }{y}}}_{.0{t}_0}\right)}^2}+\frac{{\overline{\overset{\sim }{y}}}_{.1{t}_0}^2}{\sum_{i=1}^{n_0}{\left(\tilde{y}_{{i}1{t}_0}-{\overline{\overset{\sim }{y}}}_{.1{t}_0}\right)}^2}\right){\hat{\sigma}}_{\epsilon^{(6)}}^2$ ${\hat{\sigma}}_{\epsilon^{(6)}}^2=\frac{\sum_{j=0}^1{\sum}_{i=1}^{n_j}{\left({y}_{ij{t}_1}-{\hat{y}}_{ij{t}_1}\right)}^2}{\left({n}_0+{n}_1-5\right)}$
		U	$\mathit{\operatorname{var}}\left({\hat{\beta}}_{1, ols}^{(6)}\right)=\frac{1}{n_0}\left(1-{\rho}_0^2\right){\sigma}_{01}^2+\frac{1}{n_1}\left(1-{\rho}_1^2\right){\sigma}_{11}^2+{\left({\rho}_1\frac{\sigma_{11}}{\sigma_0}-{\rho}_0\frac{\sigma_{01}}{\sigma_0}\right)}^2\frac{\sigma_0^2}{n_0+{n}_1}$	
*ANCOVA-Post* I	${\hat{\beta}}_{1, ols}^{(7)}=\left({\overline{y}}_{.1{t}_1}-{\overline{y}}_{.0{t}_1}\right)-{\hat{\beta}}_{2, ols}^{(7)}\left({\overline{y}}_{.1{t}_0}-{\overline{y}}_{.0{t}_0}\right)$	C	$\mathit{\operatorname{var}}\left({\hat{\beta}}_{1, ols}^{(7)}|{Y}_{ij{t}_0}\right)=\left(\frac{1}{n_0}+\frac{\sum \limits_{i=1}^{n_0}{\left({y}_{i1{t}_0}-{\overline{y}}_{.0{t}_0}\right)}^2\left({\overline{y}}_{.1{t}_0}-{\overline{y}}_{.0{t}_0}\right)}{\sum_{j=0}^1{\sum}_{i=1}^{n_j}{\left({y}_{ij{t}_0}-{\overline{y}}_{.j{t}_0}\right)}^2}\right)\kern0.5em {\sigma}_{\epsilon_0^{(7)}}^2+\left(\frac{1}{n_1}+\frac{\sum \limits_{i=1}^{n_1}{\left({y}_{i1{t}_0}-{\overline{y}}_{.1{t}_0}\right)}^2\left({\overline{y}}_{.1{t}_0}-{\overline{y}}_{.0{t}_0}\right)}{\sum \limits_{j=0}^1{\sum}_{i=1}^{n_j}{\left({y}_{i1{t}_0}-{\overline{\overset{\sim }{y}}}_{.1{t}_0}\right)}^2}\right)\kern0.5em {\sigma}_{\epsilon_1^{(7)}}^2$ $\kern0.5em {\sigma}_{\epsilon_0^{(7)}}^2=\left(1-{\rho}_0^2\right){\sigma}_{01}^2$, $\kern0.5em {\sigma}_{\epsilon_1^{(7)}}^2=\left(1-{\rho}_1^2\right){\sigma}_{11}^2$	${\hat{\mathit{\operatorname{var}}}}_{ols}\left({\hat{\beta}}_{1, ols}^{(7)}|{Y}_{ij{t}_0}\right)=\left(\frac{1}{n_0}+\frac{1}{n_1}+\frac{\sum \limits_{i=1}^{n_0}{\left({y}_{i1{t}_0}-{\overline{y}}_{.0{t}_0}\right)}^2\left({\overline{y}}_{.1{t}_0}-{\overline{y}}_{.0{t}_0}\right)}{\sum_{j=0}^1{\sum}_{i=1}^{n_j}{\left({y}_{ij{t}_0}-{\overline{y}}_{.j{t}_0}\right)}^2}+\frac{\sum \limits_{i=1}^{n_1}{\left({y}_{i1{t}_0}-{\overline{y}}_{.1{t}_0}\right)}^2\left({\overline{y}}_{.1{t}_0}-{\overline{y}}_{.0{t}_0}\right)}{\sum \limits_{j=0}^1{\sum}_{i=1}^{n_j}{\left({y}_{i1{t}_0}-{\overline{\overset{\sim }{y}}}_{.1{t}_0}\right)}^2}\right){\hat{\sigma}}_{\epsilon^{(7)}}^2$ ${\hat{\sigma}}_{\epsilon^{(7)}}^2=\frac{\sum_{j=0}^1{\sum}_{i=1}^{n_j}{\left({y}_{ij{t}_1}-{\hat{y}}_{ij{t}_1}\right)}^2}{\left({n}_0+{n}_1-4\right)}$
		U	$\mathit{\operatorname{var}}\left({\hat{\beta}}_{1, ols}^{(7)}\right)=\frac{1}{n_0}\left[\left(1-{\rho}_0^2\right){\sigma}_{01}^2+{\left(\left({\rho}_1\frac{\sigma_{11}}{\sigma_0}-{\rho}_0\frac{\sigma_{01}}{\sigma_0}\right){p}_1\right)}^2{\sigma}_0^2+\frac{1}{n_1}\right[\left(1-{\rho}_1^2\right)\ {\sigma}_{11}^2+{\left(\left({\rho}_1\frac{\sigma_{11}}{\sigma_0}-{\rho}_0\frac{\sigma_{01}}{\sigma_0}\right){p}_0\right)}^2{\sigma}_0^2$]	
*cRM*	${\hat{\gamma}}_{3,\kern0.5em gls}^{(4)}=\left({\overline{y}}_{.1{t}_1}-{\overline{y}}_{.0{t}_1}\right)-\Big(\frac{\rho_0{\sigma}_0{\sigma}_{01}}{\sigma_0^2}\left({\overline{y}}_{.1{t}_0}-{\overline{y}}_{..{t}_0}\right)-\frac{\rho_1{\sigma}_0{\sigma}_{11}}{\sigma_0^2}\left({\overline{y}}_{.1{t}_0}-{\overline{y}}_{..{t}_0}\right)$)	U	$\mathit{\operatorname{var}}\left({\hat{\gamma}}_{3,\kern0.5em gls}^{(4)}\right)=\frac{1}{n_0}\left[\left(1-{\rho}_0^2\right){\sigma}_{01}^2+{\left(\left({\rho}_1\frac{\sigma_{11}}{\sigma_0}-{\rho}_0\frac{\sigma_{01}}{\sigma_0}\right){p}_1\right)}^2{\sigma}_0^2+\frac{1}{n_1}\right[\left(1-{\rho}_1^2\right)\ {\sigma}_{11}^2+{\left(\left({\rho}_1\frac{\sigma_{11}}{\sigma_0}-{\rho}_0\frac{\sigma_{01}}{\sigma_0}\right){p}_0\right)}^2{\sigma}_0^2$]	

We let ${\mathit{\operatorname{var}}}_{ols}\left({\hat{\beta}}_{1, ols}^{(6)}|{\overset{\sim }{Y}}_{ij{t}_0}\right)$ denote the OLS model-based conditional variance of ${\hat{\beta}}_{1, ols}^{(6)}$ incorporating ${\sigma}_{\epsilon^{(6)}}^2$ (Table 2). Since ${\sigma}_{\epsilon^{(6)}}^2$ is generally unknown, ${\sigma}_{\epsilon^{(6)}}^2$ is estimated by

$$ {\hat{\sigma}}_{\epsilon^{(6)}}^2=\frac{\sum_{j=0}^1{\sum}_{i=1}^{n_j}{\left({y}_{ij{t}_1}-{\hat{y}}_{ij{t}_1}\right)}^2}{\left({n}_0+{n}_1-4\right)}, $$

where ${\hat{y}}_{ij{t}_1}$ is the predicted value of ${y}_{ij{t}_1}$. We let ${\hat{\mathit{\operatorname{var}}}}_{ols}\left({\hat{\beta}}_{1, ols}^{(6)}|{\overset{\sim }{Y}}_{ij{t}_0}\right)$ denote the OLS model-based variance estimator of ${\hat{\beta}}_{1, ols}^{(6)}$ with ${\hat{\sigma}}_{\epsilon^{(6)}}^2$ substituted for ${\sigma}_{\epsilon^{(6)}}^2$. and known constant ${\mu}_{t_0}$ (Table 2). ${\hat{\mathit{\operatorname{var}}}}_{ols}\left({\hat{\beta}}_{1, ols}^{(6)}|{\overset{\sim }{Y}}_{ij{t}_0}\right)$ is reported by standard statistical softwares (e.g., “proc reg” in SAS). To assess the validity of the model-based standard errors and *p*-values from a regular ***ANCOVA*II** model for unconditional inference, we need to examine: i) whether ${\hat{\mathit{\operatorname{var}}}}_{ols}\left({\hat{\beta}}_{1, ols}^{(6)}|{\overset{\sim }{Y}}_{ij{t}_0}\right)$ is unbiased for $\mathit{\operatorname{var}}\Big({\hat{\beta}}_{1, ols}^{(6)}\left|{\overset{\sim }{Y}}_{ij{t}_0}\right)$; ii) whether $\mathit{\operatorname{var}}\left({\hat{\beta}}_{1, ols}^{(6)}|{\overset{\sim }{Y}}_{ij{t}_0}\right)$ is unbiased for $\mathit{\operatorname{var}}\left({\hat{\beta}}_{1, ols}^{(6)}\right)$.

First, ${\hat{\mathit{\operatorname{var}}}}_{ols}\left({\hat{\beta}}_{1, ols}^{(6)}|{\overset{\sim }{Y}}_{ij{t}_0}\right)$ is unbiased for ${\mathit{\operatorname{var}}}_{ols}\left({\hat{\beta}}_{1, ols}^{(6)}|{\overset{\sim }{Y}}_{ij{t}_0}\right).$ However, the unbiasedness of ${\hat{\mathit{\operatorname{var}}}}_{ols}\left({\hat{\beta}}_{1, ols}^{(6)}|{\overset{\sim }{Y}}_{ij{t}_0}\right)$ as an estimator of $\mathit{\operatorname{var}}\left({\hat{\beta}}_{1, ols}^{(6)}|{\overset{\sim }{Y}}_{ij{t}_0}\right)$ depends on the relationship between ${\mathit{\operatorname{var}}}_{ols}\left({\hat{\beta}}_{1, ols}^{(6)}|{\overset{\sim }{Y}}_{ij{t}_0}\right)$ and $\mathit{\operatorname{var}}\left({\hat{\beta}}_{1, ols}^{(6)}|{\overset{\sim }{Y}}_{ij{t}_0}\right)$. Asymptotically, we have

$$ {\displaystyle \begin{array}{c}{\Delta }_{{\hat{\beta}}_{1, ols}^{(6)}}={\mathit{\operatorname{var}}}_{ols}\left({\hat{\beta}}_{1, ols}^{(6)}\left|{\overset{\sim }{Y}}_{ij{t}_0}\right)-\mathit{\operatorname{var}}\right({\hat{\beta}}_{1, ols}^{(6)}\left|{\overset{\sim }{Y}}_{ij{t}_0}\right)\\ {}=\left({\sigma}_{\epsilon_0^{(6)}}^2-{\sigma}_{\epsilon_1^{(6)}}^2\right)\left(\ \frac{1}{n_1}-\frac{1}{n_0}\right)\end{array}} $$

It can be shown in a balanced design (*n*_0_ = *n*_1_),

$$ {\mathit{\operatorname{var}}}_{ols}\left({\hat{\beta}}_{1, ols}^{(6)}\left|{\overset{\sim }{Y}}_{ij{t}_0}\right)\approx \mathit{\operatorname{var}}\right({\hat{\beta}}_{1, ols}^{(6)}\left|{\overset{\sim }{Y}}_{ij{t}_0}\right). $$

Thus, ${\hat{\mathit{\operatorname{var}}}}_{ols}\left({\hat{\beta}}_{1, ols}^{(6)}|{\overset{\sim }{Y}}_{ij{t}_0}\right)$ is nearly unbiased for $\mathit{\operatorname{var}}\Big({\hat{\beta}}_{1, ols}^{(6)}\left|{\overset{\sim }{Y}}_{ij{t}_0}\right)\ \left[3\right].$ When the design is unbalanced (*n*_0_ ≠ *n*_1_),

$$ {\mathit{\operatorname{var}}}_{ols}\left({\hat{\beta}}_{1, ols}^{(6)}\left|{\overset{\sim }{Y}}_{ij{t}_0}\right)\ne \mathit{\operatorname{var}}\right({\hat{\beta}}_{1, ols}^{(6)}\left|{\overset{\sim }{Y}}_{\mathrm{i}j{t}_0}\right). $$

Hence, ${\hat{\mathit{\operatorname{var}}}}_{ols}\left({\hat{\beta}}_{1, ols}^{(6)}|{\overset{\sim }{Y}}_{ij{t}_0}\right)$ is biased for $\mathit{\operatorname{var}}\Big({\hat{\beta}}_{1, ols}^{(6)}\left|{\overset{\sim }{Y}}_{ij{t}_0}\right).$ Due to heteroscedasticity, ${\hat{\mathit{\operatorname{var}}}}_{ols}\left({\hat{\beta}}_{1, ols}^{(6)}|{\overset{\sim }{Y}}_{ij{t}_0}\right)$ over-estimates $\mathit{\operatorname{var}}\left({\hat{\beta}}_{1, ols}^{(6)}|{\overset{\sim }{Y}}_{ij{t}_0}\right)$ if the group with a larger residual variance has larger sample size and the group with a smaller residual variance has smaller sample size, and otherwise may underestimate $\mathit{\operatorname{var}}\left({\hat{\beta}}_{1, ols}^{(6)}|{\overset{\sim }{Y}}_{ij{t}_0}\right)$ [3, 4].

Second, the common mean baseline weight ${\mu}_{t_0}$ is generally unknown. We need to estimate ${\mu}_{t_0}$ in ${\overset{\sim }{Y}}_{ij{t}_0}$ using the overall sample mean ${\hat{\mu}}_{t_0}=\frac{\sum_{j=0}^1{\sum}_{i=1}^{n_j}{Y}_{ij{t}_0}}{n_0+{n}_1}$ but ANCOVA treats ${\hat{\mu}}_{t_0}$ as fixed and fails to capture this additional variability in the conditional variances. As shown below, it turns out that $\mathit{\operatorname{var}}\left({\hat{\beta}}_{1, ols}^{(6)}|{\overset{\sim }{Y}}_{ij{t}_0}\right)$ underestimates $\mathit{\operatorname{var}}\left({\hat{\beta}}_{1, ols}^{(6)}\right)$ by a factor of ${\beta}_{3, ols}^{\left[6\right]2}\mathit{\operatorname{var}}\left({\hat{\mu}}_{t_0}\right)$ [3]:

$$ \mathit{\operatorname{var}}\left({\hat{\beta}}_{1, ols}^{(6)}\right)=E\left(\mathit{\operatorname{var}}\left({\hat{\beta}}_{1, ols}^{(6)}|{\overset{\sim }{Y}}_{ij{t}_0}\right)\right)+{\beta}_{3, ols}^{(6)2}\mathit{\operatorname{var}}\left({\hat{\mu}}_{t_0}\right). $$

Thus, the OLS model-based conditional inference is biased for unconditional hypothesis testing because of heteroscedasticity and neglecting of sampling variability in ${\hat{\mu}}_{t_0}$. To fix these two problems, we can use the following adjusted heteroscedasticity-consistent (HC) variance estimator to replace ${\hat{\mathit{\operatorname{var}}}}_{ols}\left({\hat{\beta}}_{1, ols}^{(6)}|{\overset{\sim }{Y}}_{ij{t}_0}\right)$ for valid unconditional inference:

$$ {\hat{\mathit{\operatorname{var}}}}_{aHC}\left({\hat{\beta}}_{1, ols}^{(6)}\left|{\overset{\sim }{Y}}_{ij{t}_0}\right)={\hat{\mathit{\operatorname{var}}}}_{HC}\right({\hat{\beta}}_{1, ols}^{(6)}\left|{\overset{\sim }{Y}}_{ij{t}_0}\right)+{\hat{\beta}}_{3, ols}^{(6)2}\frac{{\hat{\sigma}}_0^2}{n_0+{n}_1}, $$

where ${\hat{\mathit{\operatorname{var}}}}_{HC}\Big({\hat{\beta}}_{1, ols}^{(6)}\left|{\overset{\sim }{Y}}_{ij{t}_0}\right)$ is a HC variance estimator for $\mathit{\operatorname{var}}\left({\hat{\beta}}_{1, ols}^{(6)}|{\overset{\sim }{Y}}_{ij{t}_0}\right)$ [19] and can be output from standard softwares. HC variance estimators are consistent (i.e., unbiased in large sample). Among all available HC variance estimators, HC2 was shown to have the best performance in finite samples [3, 4] (e.g. “HCCMETHOD = 2” in proc. reg or “EMPIRICAL” in proc. mixed, SAS). ${\hat{\beta}}_{3, ols}^{\left[6\right]}$ is the OLS estimator of ${\beta}_3^{(6)}$, and ${\hat{\sigma}}_0^2$ is the overall sample variance of the baseline body weight. It follows directly that ${\hat{\mathit{\operatorname{var}}}}_{aHC}\left({\hat{\beta}}_{1, ols}^{(6)}|{\overset{\sim }{Y}}_{ij{t}_0}\right)$ is asymptotically unbiased for $\mathit{\operatorname{var}}\left({\hat{\beta}}_{1, ols}^{(6)}\right)$ and we can construct a valid test $t=\frac{{\hat{\beta}}_{1, ols}^{(6)}}{\sqrt{{\hat{\mathit{\operatorname{var}}}}_{aHC}\left({\hat{\beta}}_{1, ols}^{(6)}|{\overset{\sim }{Y}}_{ij{t}_0}\right)}\ }$ for testing *H*_*o*_ : *τ* = 0 unconditionally.

**Method 7*ANCOVA*I:** We model the post-treatment weight ${Y}_{ij{t}_1}$ using the binary treatment *G* and the baseline weight ${Y}_{ij{t}_0}$:

7 $$ {Y}_{ij{t}_1}={\beta}_0^{(7)}+{\beta}_1^{(7)}{G}_{ij}+{\beta}_2^{(7)}{Y}_{ij{t}_0}+{e}_{ij}^{(7)} $$

$$ {e}_{i0}^{(7)}\sim N\left(0,\kern0.5em {\sigma}_{\epsilon_0^{(7)}}^2\right)\ \mathrm{and}\kern0.75em {\sigma}_{\epsilon_0^{(7)}}^2=\left(1-{\rho}_0^2\right){\sigma}_{01}^2+{\left({\beta}_3^{(6)}{p}_1\right)}^2{\sigma}_0^2 $$

$$ {e}_{i1}^{(7)}\sim N\left(0,\kern0.5em {\sigma}_{\epsilon_1^{(7)}}^2\right)\ \mathrm{and}\kern0.75em {\sigma}_{\epsilon_1^{(7)}}^2=\left(1-{\rho}_1^2\right){\sigma}_{11}^2+{\left({\beta}_3^{(6)}{p}_0\right)}^2{\sigma}_0^2 $$

, where ${\beta}_0^{(7)}={\beta}_0^{(6)}-{\beta}_3^{(6)}{p}_0{\mu}_0,$ and ${\beta}_1^{(7)}=\tau$. ${e}_{i0}^{(7)}$ and ${e}_{i1}^{(7)}$ are random errors in the control and treatment arms. Since ${e}_{i0}^{(7)}$ and ${e}_{i1}^{(7)}$ have different variances in general, model (7) is heteroscedastic and the severity of heteroscedasticity is determined by the correlation coefficient, the variances of the post-treatment weights in two arms, and whether the design is balanced.

As shown in Table 2, the OLS estimator ${\hat{\beta}}_{1, ols}^{(7)}$ is an adjusted mean difference in the post-treatment weights controlling for a weighted mean difference of the baseline weights between two arms with equal weighting coefficient for the treatment and control arms (i.e., ${\hat{\beta}}_{2, ols}^{(7)}$ for both arms). ${\hat{\beta}}_{1, ols}^{(7)}$ is unbiased for *τ*. The true conditional variance $\mathit{\operatorname{var}}\left({\hat{\beta}}_{1, ols}^{(7)}|{Y}_{ij{t}_0}\right)$ incorporates two different residual variances. Similar to ***ANCOVA*II**, the OLS model-based inference for ***ANCOVA*I** also mistakenly assumes a constant residual variance ${\sigma}_{\epsilon^{(7)}}^2$, which is a weighted average of $\kern0.5em {\sigma}_{\epsilon_0^{(7)}}^2$ and $\kern0.5em {\sigma}_{\epsilon_1^{(7)}}^2$, as follows:

$$ {\sigma}_{\epsilon^{(7)}}^2=\frac{n_0}{n_0+{n}_1}\kern0.5em {\sigma}_{\epsilon_0^{(7)}}^2+\frac{n_1}{n_0+{n}_1}{\sigma}_{\epsilon_1^{(7)}}^2. $$

Since ${\sigma}_{\epsilon^{(7)}}^2$ is unknown, it is estimated by

$$ {\hat{\sigma}}_{\epsilon^{(7)}}^2=\frac{\sum_{j=0}^1{\sum}_{i=1}^{n_j}{\left({y}_{ij{t}_1}-{\hat{y}}_{ij{t}_1}\right)}^2}{n_0+{n}_1-3}, $$

where ${\hat{y}}_{ij{t}_1}$ is the predicted value of ${y}_{ij{t}_1}$ from model (7). The closed form expressions of the OLS model-based conditional variance ${\mathit{\operatorname{var}}}_{ols}\left({\hat{\beta}}_{1, ols}^{(7)}|{Y}_{ij{t}_0}\right)$ incorporating ${\sigma}_{\epsilon^{(7)}}^2$ and the OLS model-based variance estimator ${\hat{\mathit{\operatorname{var}}}}_{ols}\left({\hat{\beta}}_{1, ols}^{(7)}|{Y}_{ij{t}_0}\right)$ with ${\hat{\sigma}}_{\epsilon^{(7)}}^2$ substituted for ${\sigma}_{\epsilon^{(7)}}^2$ are given in Table 2. Recall that standard statistical softwares report ${\hat{\mathit{\operatorname{var}}}}_{ols}\left({\hat{\beta}}_{1, ols}^{(7)}|{Y}_{ij{t}_0}\right)$. To show the model-based standard errors and *p*-values are valid for unconditional inference, we need to examine: i) whether ${\hat{\mathit{\operatorname{var}}}}_{ols}\left({\hat{\beta}}_{1, ols}^{(7)}|{Y}_{ij{t}_0}\right)$ is unbiased for $\mathit{\operatorname{var}}\Big({\hat{\beta}}_{1, ols}^{(7)}\left|{Y}_{ij{t}_0}\right)$; ii) whether $\mathit{\operatorname{var}}\left({\hat{\beta}}_{1, ols}^{(7)}|{Y}_{ij{t}_0}\right)$ is unbiased for $\mathit{\operatorname{var}}\left({\hat{\beta}}_{1, ols}^{(7)}\right)$.

First, ${\hat{\mathit{\operatorname{var}}}}_{ols}\left({\hat{\beta}}_{1, ols}^{(7)}|{Y}_{ij{t}_0}\right)$ is unbiased for ${\mathit{\operatorname{var}}}_{ols}\left({\hat{\beta}}_{1, ols}^{(7)}|{Y}_{ij{t}_0}\right)$ but the unbiasedness of ${\hat{\mathit{\operatorname{var}}}}_{ols}\left({\hat{\beta}}_{1, ols}^{(7)}|{Y}_{ij{t}_0}\right)$ as an estimator of $\mathit{\operatorname{var}}\left({\hat{\beta}}_{1, ols}^{(7)}|{Y}_{ij{t}_0}\right)$ depends on the relationship between ${\mathit{\operatorname{var}}}_{ols}\left({\hat{\beta}}_{1, ols}^{(7)}|{Y}_{ij{t}_0}\right)$ and $\mathit{\operatorname{var}}\left({\hat{\beta}}_{1, ols}^{(7)}|{Y}_{ij{t}_0}\right)$. Asymptotically, we have

$$ {\displaystyle \begin{array}{c}{\Delta }_{{\hat{\beta}}_{1, ols}^{(7)}}={\mathit{\operatorname{var}}}_{ols}\Big({\hat{\beta}}_{1, ols}^{(7)}\left|{Y}_{ij{t}_0}\right)-\mathit{\operatorname{var}}\left({\hat{\beta}}_{1, ols}^{(7)}|{Y}_{ij{t}_0}\right)\\ {}=\left({\sigma}_{\epsilon_0^{(7)}}^2-{\sigma}_{\epsilon_1^{(7)}}^2\right)\left(\ \frac{1}{n_1}-\frac{1}{n_0}\right)\end{array}} $$

When sample sizes are equal between two arms, we have

$$ {\mathit{\operatorname{var}}}_{ols}\left({\hat{\beta}}_{1, ols}^{(7)}\left|{Y}_{ij{t}_0}\right)\approx \mathit{\operatorname{var}}\right({\hat{\beta}}_{1, ols}^{(7)}\left|{Y}_{ij{t}_0}\right). $$

Thus, ${\hat{\mathit{\operatorname{var}}}}_{ols}\left({\hat{\beta}}_{1, ols}^{(7)}|{Y}_{ij{t}_0}\right)$ is nearly unbiased for $\mathit{\operatorname{var}}\left({\hat{\beta}}_{1, ols}^{(7)}|{Y}_{ij{t}_0}\right)$ in a balanced design [3]. When sample sizes are not equal between two arms,

$$ {\mathit{\operatorname{var}}}_{ols}\left({\hat{\beta}}_{1, ols}^{(7)}\left|{Y}_{ij{t}_0}\right)\ne \mathit{\operatorname{var}}\right({\hat{\beta}}_{1, ols}^{(7)}\left|{Y}_{ij{t}_0}\right), $$

it follows directly that ${\hat{\mathit{\operatorname{var}}}}_{ols}\left({\hat{\beta}}_{1, ols}^{(7)}|{Y}_{ij{t}_0}\right)$ is biased for $\mathit{\operatorname{var}}\left({\hat{\beta}}_{1, ols}^{(7)}|{Y}_{ij{t}_0}\right)$ due to heteroscedasticity. ${\hat{\mathit{\operatorname{var}}}}_{ols}\left({\hat{\beta}}_{1, ols}^{(7)}|{Y}_{ij{t}_0}\right)$ may over-estimate $\mathit{\operatorname{var}}\left({\hat{\beta}}_{1, ols}^{(7)}|{Y}_{ij{t}_0}\right)$ when the group with a larger residual variance has larger sample size and the group with a smaller residual variance has smaller sample size, and otherwise may underestimate $\mathit{\operatorname{var}}\left({\hat{\beta}}_{1, ols}^{(7)}|{\overset{\sim }{Y}}_{ij{t}_0}\right)$ [3, 4] . ***ANCOVA*I** is robust against heteroscedasticity in a balanced design, but not in an unbalanced design.

Second, different from ***ANCOVA*II**, $\mathit{\operatorname{var}}\left({\hat{\beta}}_{1, ols}^{(7)}|{Y}_{ij{t}_0}\right)$ is unbiased for $\mathit{\operatorname{var}}\left({\hat{\beta}}_{1, ols}^{(7)}\right)$ because $\mathit{\operatorname{var}}\left({\hat{\beta}}_{1, ols}^{(7)}\right)=E\left(\mathit{\operatorname{var}}\left({\hat{\beta}}_{1, ols}^{(7)}|{Y}_{ij{t}_0}\right)\right)$.

Thus, the model-based standard errors and *p*-values are valid for unconditional inference in a balanced design but are biased in an unbalanced design only due to heteroscedasticity. This bias can be easily corrected by replacing ${\hat{\mathit{\operatorname{var}}}}_{ols}\left({\hat{\beta}}_{1, ols}^{(7)}|{Y}_{ij{t}_0}\right)$ with an HC variance estimator ${\hat{\mathit{\operatorname{var}}}}_{HC}\left({\hat{\beta}}_{1, ols}^{(7)}|{Y}_{ij{t}_0}\right)$[4, 19] and corrected ***ANCOVA*I** will provide valid unconditional inference.

***Constrained Repeated Measures heterogeneous variance model (“cRM”)*:** We model the baseline and post-treatment weights ($$ {Y}_{ij{t}_0}, $$$$ {Y}_{ij{t}_1} $$) jointly using the binary time point *T*_*ij*_, time by treatment interaction *G*_*ij*_ × *T*_*ij*_:

8 $$ {Y}_{ij t}={\gamma}_0^{(8)}+{\gamma}_1^{(8)}{T}_{ij}+{\gamma}_2^{(8)}{G}_{ij}\times {T}_{ij}+{e}_{ij t}^{(8)}\ j=0,1;i=1,2,\dots {n}_j. $$

$$ \left(\begin{array}{c}{e}_{i0{t}_0}^{(8)}\\ {}{e}_{i0{t}_1}^{(8)}\end{array}\right)\sim N\left(\left[\begin{array}{c}0\\ {}0\end{array}\right],{\sum}_0\right)\ \mathrm{in}\ \mathrm{the}\ \mathrm{control}\ \mathrm{arm}, $$

$$ \left(\begin{array}{c}{e}_{i1{t}_0}^{(8)}\\ {}{e}_{i1{t}_1}^{(8)}\end{array}\right)\sim N\left(\left[\begin{array}{c}0\\ {}0\end{array}\right],{\sum}_1\right)\ \mathrm{in}\ \mathrm{the}\ \mathrm{treatment}\ \mathrm{arm}, $$

where ${\gamma}_0^{(8)}={\mu}_{t_0},{\gamma}_2^{(8)}={\mu}_{0{t}_1}-{\mu}_{0{t}_0}$, and ${\gamma}_2^{(8)}=\tau$. Noting that subjects in the treatment and control arms have different variance-covariance structures for the association between the pre- and post-treatment weights, we fit a ***cRM*** heterogeneous variance GLS model with group specific variance-covariance structure (“repeated/group=” in SAS proc. mixed procedure specifies distinct variance-covariance structure for each treatment arm). The formulas of ${\hat{\gamma}}_{2, gls}^{(8)}$ and $\mathit{\operatorname{var}}\left({\hat{\gamma}}_{2, gls}^{(8)}\right)$are listed in Table 2. The GLS estimator ${\hat{\gamma}}_{2, gls}^{(8)}$is asymptotically unbiased for${\gamma}_2^{(8)}$. REML is used to derive the empirical or model-based variance estimator${\hat{\ \mathit{\operatorname{var}}}}_{reml}\Big({\hat{\gamma}}_{2,\kern0.5em gls}^{(8)}$).

## Results

All treatment effect estimators, except the ANOVA estimator, are expressed as the mean difference in post-treatment measurements adjusting for the chance imbalance in baseline measurement between two arms in certain ways. Nonetheless, all estimators are unbiased for *τ*. To compare these competing methods, we evaluate the efficiency of point estimators of treatment effect by comparing their “unconditional” variances. Since the hypothesis testing of no treatment effect is based on dividing the point estimator by its standard error (i.e., variance divided by sample size) and rejecting the null hypothesis when this ratio exceeds a given threshold, the method that produces unbiased point estimate with the smallest unconditional variance is preferred because standard error in the dominator of statistical test determines the statistical power.

### When study population is homogeneous

***ANCOVA*I** is a more efficient alternative to ***ANOVA*** because $$ \mathit{\operatorname{var}}\left({\hat{\beta}}_{1, ols}^{(2)}\right)\le \mathit{\operatorname{var}}\left({\hat{\beta}}_{1, ols}^{(1)}\right) $$ (Table 1). This advantage of ANCOVA over ANOVA can also be observed from the fact that the residual error variance of ***ANCOVA*I** is less than the residual error variance of ***ANOVA*** (i.e.,$$ \left(1-{\rho}^2\right){\sigma}_1^2 $$$$ \le {\sigma}_1^2 $$). When the correlation coefficient *ρ* becomes larger, the ***ANCOVA*I** estimator has smaller variance. Since $$ {Y}_{ij{t}_1} $$and $$ {Y}_{ij{t}_0} $$are highly correlated in general, the inclusion of $$ {Y}_{ij{t}_0} $$ in ***ANCOVA*I** explains away some variability in$$ {Y}_{ij{t}_1} $$ and thus reduces the residual variance and yields a more efficient estimator of treatment effect than ***ANOVA***.

***ANOVA-Change*** and ***RM*** have exactly same point estimators of *τ* and thus have the same variances (Table 1). To compare ***ANOVA-Change*** or ***RM*** with ***ANOVA***, we can derive the difference between the unconditional variances of their treatment effect estimators as follows:

$$ {\Delta }_1={\sigma}_0\left(1-2\rho {\sigma}_1\right). $$

When $\rho <\frac{1}{2{\sigma}_1}$, ∆_1_ > 0 and ***ANOVA*** outperforms ***ANOVA-Change*** and ***RM*** because the ***ANOVA*** estimator has smaller variance**.** When $\rho >\frac{1}{2{\sigma}_1}$, ∆_1_ < 0 and ***ANOVA*** underperforms the other two methods.

It can be shown that the difference between the unconditional variances of the ***ANCOVA*I** or ***cRM*** estimators and those of **the*ANOVA-Change*** or ***RM*** estimators are always nonnegative:

$$ {\displaystyle \begin{array}{c}{\Delta }_2=\left({\sigma}_1^2+{\sigma}_0^2-2\rho {\sigma}_0{\sigma}_1\right)-\left(1-{\rho}^2\right){\sigma}_1^2\\ {}={\left({\sigma}_0-{\rho \sigma}_1\right)}^2\ge 0\end{array}} $$

Thus, ***ANOVA-Change*** or ***RM*** is less efficient than either ***ANCOVA*I** or ***cRM*** because their estimators have larger variances. Intuitively ***ANCOVA*I** or ***cRM*** assumes that mean baseline weights in two arms are equal in a randomized study but ***ANOVA-Change*** or ***RM*** assumes that there is a baseline difference and needs to estimate an extra parameter.

As shown in Table 1, the ***ANCOVA*I** and ***cRM*** estimators of *τ* are equivalent because ${\beta}_{1, ols}^{(2)}$ = $\frac{\rho {\sigma}_0{\sigma}_1}{\sigma_0^2}$. However, ***ANCOVA*I** plugs in the OLS estimators${\hat{\beta}}_{1, ols}^{(2)}$, whereas ***cRM*** plugs in the REML estimators of the variance and covariance parameters. The numerical difference between ${\hat{\beta}}_{1, ols}^{(2)}$ and ${\hat{\gamma}}_{3, gls}^{(4)}$ becomes negligible as sample size increases. Because of this equivalence between ${\hat{\beta}}_{1, ols}^{(2)}$ and ${\hat{\gamma}}_{3, gls}^{(4)}$,$\mathit{\operatorname{var}}\left({\hat{\beta}}_{1, ols}^{(2)}\right)$ and $\mathit{\operatorname{var}}\left({\hat{\gamma}}_{3,\kern0.5em gls}^{(4)}\right)$ are equal [3]. As discussed previously, ***ANCOVA*I** is a conditional model assuming fixed baseline covariates. Even though the model-based variance estimates are conditional, they are unbiased for the unconditional variance and thus the usual model-based conditional inference is still valid for unconditional hypothesis testing. ***ANCOVA*I** performs comparably to ***cRM*** [3, 17]**.**

### When study population is heterogeneous

A heterogeneous study population justifies the inclusion of a treatment by baseline weight interaction term. Thus, ***ANCOVA*II** is the correctly specified model, whereas ***ANCOVA*I** is a mis-specified model. In this case, the “conditional” treatment effect is not constant across different values of baseline weight. The “marginal” treatment effect *τ* is simply the average of the conditional treatment effect over the distribution of the baseline weight and measures an overall treatment effect. As shown previously, both ANCOVA models can be used to estimate *τ* even though ***ANCOVA*I** is mis-specified. Then, what is the advantage of using a more complex interaction model over a main effect model? It turns out the ***ANCOVA*II** estimator ${\hat{\beta}}_{1, ols}^{(6)}$ is more efficient than the ***ANCOVA*I** estimator ${\hat{\beta}}_{1, ols}^{(7)}$ because $\mathit{\operatorname{var}}\left({\hat{\beta}}_{1, ols}^{(6)}\right)\le \mathit{\operatorname{var}}\left({\hat{\beta}}_{1, ols}^{(7)}\right)$ [5]. Only in a balanced design $\mathit{\operatorname{var}}\left({\hat{\beta}}_{1, ols}^{(6)}\right)=\mathit{\operatorname{var}}\left({\hat{\beta}}_{1, ols}^{(7)}\right)$ and the two ANCOVA models perform comparably. Note that the OLS model-based variance estimates for ***ANCOVA*I** and **II** are both biased for the corresponding unconditional variances, but the HC-variance estimators provide simple fixes.

The ***ANCOVA*II** and ***cRM*** estimators of *τ* are equivalent because ${\beta}_2^{(6)}+{\beta}_3^{(6)}=\frac{\rho_0{\sigma}_0{\sigma}_{01}}{\sigma_0^2}$ and ${\beta}_2^{(6)}=\frac{\rho_1{\sigma}_0{\sigma}_{11}}{\sigma_0^2}$ (Table 2). Two methods only differ in the way two estimators are estimated. ***ANCOVA*II** plugs in the OLS estimators${\hat{\beta}}_{2, ols}^{(6)}$ and ${\hat{\beta}}_{3, ols}^{(6)}$, whereas ***cRM*** plugs in the REML estimators of the variance and covariance parameters. The numerical difference between the ***ANCOVA*II** and ***cRM*** estimators becomes smaller as sample size increases. As discussed previously, standard statistical softwares such as SAS does not output unconditional variance for ***ANCOVA*II** directly but the usual OLS model-based standard errors and *p*-values are biased for unconditional inference in heterogeneous scenario. The adjusted HC-variance estimator fixes this bias. Corrected ***ANCOVA*II** provides valid unconditional inference and performs comparably to ***cRM*.** Another alternative approach to estimate variances of the ***ANCOVA*I and II** estimators is to use bootstrap method [20].

### Data example

No human data was used in this study. Instead we simulated three weight loss trial data sets based on a published study for three scenarios: homogeneous data, heterogeneous data with balanced and unbalanced designs as follows [21]:

1. The baseline weights for the control and treatment arms were generated from normal distribution with mean 88 kg and standard deviation 14 kg. Weights at 6 month after treatment for the control arm have mean 86 kg and standard deviation 15 kg. This gives a ~ 2.3% change from baseline. The mean and standard deviation of body weight at the sixth month in the treatment arm are 83 kg and 15 kg, respectively; This corresponds to a 5.7% change from baseline.
2. In the homogeneous data, the correlation coefficient between the pre- and post-treatment weights is 0.9. One hundred eighty subjects were assigned to the treatment and control arms equally. In the heterogeneous data, the correlation coefficient between the pre- and post-treatment weights in the control arm is 0.9 and 0.7 in the treatment arm. Sample sizes are (*n*_0_ = 90, *n*_1_ = 90) for the balanced design and (*n*_0_ = 60, *n*_1_ = 120) for the unbalanced design. We analyzed the data examples using the methods outlined in section Methods. The statistical results were reported in Table 3 (SAS programs are provided in the Additional file 1).

**Table 3:** Statistical analysis of the three simulated data examples

Scenario	Method	Estimate	Standard error	*p-value*
**Homogeneous**	***ANOVA***	−3.089	2.106	0.144
	***ANCOVA*I**	−2.422	0.955	0.0121
	***ANOVA-Change***	−2.354	0.971	0.0163
	***RM***	−2.354	0.971	0.0163
	***cRM***	−2.434	0.944	0.0108
**Heterogeneous** **(***n*_0_ = 90, *n*_1_ = 90**)**	***ANCOVA*I**	−3.203	1.403^a^	0.0235
			1.397^b^	0.0231
			1.400^d^	n/a
	***ANCOVA*II**	−3.165	1.333^a^	0.0187
			1.402^c^	0.0252
			1.397^d^	n/a
	***cRM***	−3.203	1.405	0.0241
**Heterogeneous** (*n*_0_ = 60, *n*_1_ = 120)	***ANCOVA*I**	−3.416	1.415^a^	0.0167
			1.279^b^	0.0083
			1.281^d^	n/a
	***ANCOVA*II**	−3.399	1.376^a^	0.0145
			1.258^c^	0.0076
			1.260^d^	n/a
	***cRM***	−3.396	1.262	0.0078

^a^OLS regression model-based standard error

^b^HC standard error for ANCOVA I (main effect) model

^c^Modified HC standard error for ANCOVA II (interaction) model

^d^Bootstrapping standard error (*n* = 5000)

In the first data example, ***ANOVA*** produced the largest standard error and the largest *p*-value. ***ANOVA-Change*** and ***RM*** both outperformed ***ANOVA*** with much smaller standard errors and *p*-values. ***ANCOVA*I** and ***cRM*** outperformed ***ANOVA-Change*** and ***RM*** with smaller standard errors and *p*-values. Although ***ANCOVA*I** and ***cRM*** are equivalent when sample size is large, there are still minor numerical differences between the two in finite sample.

**Fig. 2:**
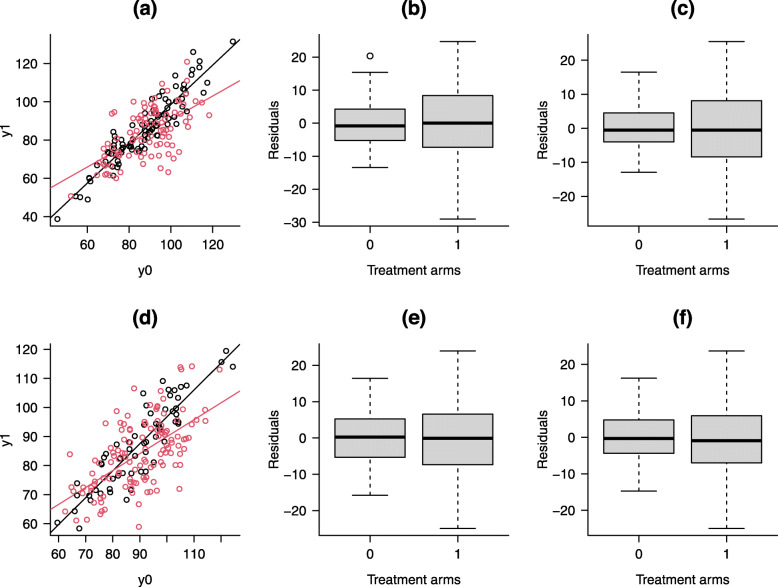
Diagnosis plots of ANCOVA main and interaction models in heterogeneous scenario. **a** Scatter plot of baseline and follow-up weights in balanced design. Black and red solid dots are data points in the treatment and control arms. Black and red solid lines are the regression slopes of baseline weight against follow-up weight in the treatment and control arms. **b** Boxplot of residuals from the treatment and control arms from ***ANCOVA*I** model in balanced design; **c** Boxplot of residuals from the treatment and control arms from ***ANCOVA*II** model in balanced design; **d** Scatter plot of baseline and follow-up weights in unbalanced design. Black and red solid dots are data points in the treatment and control arms. Black and red solid lines are the regression slopes of baseline weight against follow-up weight in the treatment and control arms. **e** Boxplot of residuals from the treatment and control arms from ***ANCOVA*I** model in an unbalanced design; **f** Boxplot of residuals from the treatment and control arms from ***ANCOVA*II** model in an unbalanced design

For the second data example with a balanced design, Fig. 2a shows that there is a strong baseline weight by treatment interaction. Both ***ANCOVA*I** and **II** have heteroscedastic errors by treatment arm (Fig. 2b and c). As shown in Table 2, the OLS model-based standard error of ***ANCOVA*I** is very similar to its HC and bootstrap standard errors. Thus, heteroscedasticity does not bias the model-based standard error of ***ANCOVA*I**. Although ***ANCOVA*II** is robust against heteroscedasticity in the balanced design, the OLS model-based standard error of ***ANCOVA*II** (*s.e* = 1.333) is still not correct because OLS fails to consider the variability of estimating the overall mean baseline weight. The adjusted HC standard error for ***ANCOVA*II** is 1.402, which is closer to the model-based and HC standard errors of ***ANCOVA*I**. The bootstrapping standard errors for ***ANCOVA*I** and **II** are close to their HC or adjusted HC standard errors, which suggests the HC and adjusted HC variances perform well in estimating the unconditional variances. The ***cRM*** estimate and its standard error are close to those from ***ANCOVA*I** and **II**.

For the third example with an unbalanced design, Fig. 2d also reveals a baseline weight by treatment interaction. Both ANCOVA models have heteroscedastic errors by treatment arm (Fig. 2e and f). The model-based standard errors of ***ANCOVA*I** and **II** are not valid. The model-based standard errors were larger than the HC standard errors and thus overestimated the true conditional variances. Compared with ***ANCOVA*I**, ***ANCOVA*II** has a smaller HC standard error (also smaller *p*-value) and thus is slightly more efficient. The adjusted HC standard error for ***ANCOVA*II** is very close to the model-based standard error for ***cRM***. The bootstrapping standard errors for ***ANCOVA*I** and **II** are very close to their HC or adjusted HC standard errors.

## Discussion

In this study we compare the efficiency of six unbiased methods analyzing pre-post designs. We found ANCOVA and ***cRM*** are the equally most efficient methods compared with other alternatives in homogeneous and heterogeneous scenarios. In this study, we focus on the scenario in which randomization is properly performed and these competing methods all target the same causal quantity. In the scenarios where the treatment is not properly randomized or not randomized at all (e.g., in an observational study), the baseline score will not be balanced by design. In this case these competing methods may target different causal quantities. Debate over using change-score analysis (or RM) verse ANCOVA in the non-randomized setting, generally known as the lord’s paradox, is a well-known example [22, 23].

The majority of previous studies has only examined homogeneous study population. In this setting, ***ANOVA*** is one of the least efficient approaches for analyzing pre-post designs because it does not utilize any baseline information. ***ANOVA-Change*** and ***RM*** incorporate the baseline score as part of outcome, whereas ***ANCOVA*I** includes the baseline score as a covariate. ***ANCOVA*I** outperforms ***ANOVA-Change*** and ***RM*** because ***ANCOVA*I** utilizes the assumption that the baseline scores are balanced between two arms in a randomized study. Thus, change score is a less efficient way to utilize the baseline score than including the baseline score as a covariate. Since we seldom can control the values of the baseline score in randomized trials, the OLS assumption that the baseline score is fixed casts doubt on the validity of ANCOVA for hypothesis testing [6, 12]. Crager proved ***ANCOVA*I** is valid for unconditional inference in homogeneous scenario [6]. This conclusion can be simply attributed to that the conditional variance of the ***ANCOVA*I** estimator is an unbiased estimate for its unconditional variance [3].

A few studies investigated further a heterogeneous scenario [3, 4, 10, 12, 24]. Although the heterogeneity justifies the inclusion of the baseline measurement by treatment interaction term, ***ANCOVA*I** and **II** are both unbiased. Yang and Tsiatis showed that ***ANCOVA*II** has a smaller unconditional variance estimator than that of ***ANCOVA*I** unless in a balanced design [9]. However, the OLS model-based variances of the ***ANCOVA*I** and **II** estimators, reported by standard statistical softwares, are conditional variances, not unconditional variances. The OLS model-based standard errors and associated *p*-values for ***ANCOVA*II** are generally questionable for unconditional inference, and the model-based inference for ***ANCOVA*I** is biased only when the design is unbalanced [3, 4, 10, 24]. With the corrected HC variance estimators, both models provide valid unconditional inference. Choosing between ***ANCOVA*I** and **II** then becomes an evaluation of a trade-off between simplicity and some gains in efficiency.

In homogenous setting, ***cRM*** was suggested as a superior choice to ***ANCOVA*I** because the unconditional variance of the ***cRM*** estimator is smaller than the conditional variance of the ***ANCOVA*I** estimator [25]. Kenward et al. pointed out that such direct comparison between the conditional and unconditional variances is not meaningful. Since both estimators are equivalent, it can be shown that ***cRM*** coupled with REML and Kenward-roger adjustment performs almost identically to ***ANCOVA*I** in finite samples [17]. In heterogeneous scenario, ***cRM*** is comparable to ***ANCOVA*II** [3]. In presence of missing data, applied researchers often prefer ***cRM*** over ANCOVA because it can utilize all observed data but ANCOVA uses only complete cases**.** However**,** imputation methods which utilize the strong pre-post correlation, such as weighting and regression imputation, can improve the statistical power for ANCOVA without biasing estimates, making it comparable to ***cRM*** [17].

Furthermore, ANCOVA has several advantages over ***cRM***: first, outcome should only be the variable that can be influenced by treatment. Baseline measurement is certainly not an outcome by this definition. It is conceptually more appropriate to include the baseline score as covariate, not model it as outcome [5]; Second, it is very convenient to include other baseline variables in a regression model for more efficient estimates of treatment effect. Third, it is easy to adjust for other patterns of heteroscedastic errors in an OLS regression. For example, we may expect larger variability in the post-treatment weights associated with larger baseline weights. ***cRM*** cannot handle this more complex type of heteroscedasticity easily. HC-variance estimators for ANCOVA are simple fixes and readily implemented in statistical softwares.

## Conclusion

Comparing with other alternative methods, ANCOVA is a simple and the most efficient approach analyzing a pre-post randomized design. When there exists a baseline score by treatment interaction, we need to assess the heteroscedasticity of ANCOVA particularly when the design is not balanced. The HC-variances should be used for valid inference when heteroscedasticity is present. Adding an interaction term in ANCOVA can gain some efficiency but not including this term does not bias results.

## Supplementary Information

**Additional file 1.**

## Data Availability

SAS code is provided as the Additional file 1. There is no real data used. All data generated or analyzed during this study are included in this published article [and its supplementary information files].

## Abbreviations

***ANOVA***: Analysis of variance model
***ANCOVA*****I**: Analysis of covariance model adjusting for the baseline measurement
***ANCOVA*****II**: Analysis of covariance model adjusting for the baseline measurement by treatment interaction
***RM***: Constrained repeated measure model
***cRM***: Constrained repeated measure model
HC: Heteroscadascity-consistent
