# Supplementary material for: Statistical analysis of two arm randomized pre-post designs with one post-treatment measurement
Source: BMC Med Res Methodol. 2021 Jul 24;21:150. doi: 10.1186/s12874-021-01323-9 (PMC8305561; doi:10.1186/s12874-021-01323-9)
Supplement: Supplementary file 1 — Additional file 1. [file 12874_2021_1323_MOESM1_ESM.zip › Supplemental material_revised.docx]

**Supplemental material for Statistical analysis of two arm randomized pre-post design with one post-treatment measurement**

Fei Wan,^1^ PhD

^1^Division of Public Health Sciences, Department of Surgery

Washington University School of Medicine

Saint Louis, MO, USA

**Corresponding Author:** Fei Wan

Division of Public Health Sciences, Department of Surgery

Washington University School of Medicine

Campus Box 8100

660 S. Euclid Ave, St. Louis, MO, USA

Tel: +1-314-362-9647; Fax: +1-314-747-3935

E-mail: [wan.fei@wustl.edu](mailto:wan.fei@wustl.edu)

SAS programs for ANCOVA I, ANCOVA II, and cRM in a heterogeneous scenario

***compute the overall mean of baseline measurement***;

**proc** **means** data=exampledata mean std stderr;

var y0;

output out=meandata mean=y0bar stderr=y0err;

**run**;

***create mean centered baseline variable y0cen***;

**data** centeredData;

set exampledata;

y0cen=y0-**90.0817872**;

int_y0cen=trt*y0cen;

**run**;

***ANCOVA I including main effect of treament and baseline variable y0***;

***use PROC REG/HHMETHOD=2 or PROC MIXED/empirical to compute HC2 variance***;

**proc** **reg** data= centeredData;

model y1=trt y0/white HCCMETHOD=**2**;

**run**;

**proc mixed** data= centeredData empirical;

model y1=trt y0/solution;

repeated /subject=subj;

run;

***ANCOVA II including treatment, y0cen, and the interaction term***;

***use HHMETHOD=2 to compute HC2 variance***;

**proc** **reg** data= centeredData;

ods output ParameterEstimates=perobust;

model y1=trt y0cen int_y0cen/white HCCMETHOD=**2**;

**run**;

**data** perobust2;

set perobust;

keep variable estimate stderr tvalue probt hcstderr;

**run**;

**proc** **sql** noprint;

select y0bar into: y0bar

from meandata;

select y0err into: y0err

from meandata;

%put &y0bar &y0err;

**proc** **sql** noprint;

select estimate into: beta0

from perobust2

where variable="Intercept";

select estimate into: beta1

from perobust2

where variable="trt";

select estimate into: beta2

from perobust2

where variable="y0cen";

select estimate into: beta3

from perobust2

where variable="int_y0cen";

**data** perobust3;

set perobust2;

if variable="trt" then modified_hcstderr=sqrt(hcstderr****2**+((&beta3)****2**)*((&y0err)****2**));

corrZ=estimate/modified_hcstderr;

pvalue=**2***(**1**-probnorm(abs(corrZ)));

if variable in ("Intercept","trt") then pvalue2=**2***(**1**-probt(abs(corrz),**176**));

if variable="trt" ;

keep variable estimate stderr tvalue probt hcstderr modified_hcstderr corrz pvalue pvalue2;

**run**;

**data** mixeddata;

set exampledata;

do; y=y1;time=**1**;time2=time;output;end;

do; y=y0;time=**0**;time2=time;output;end;

**run**;

title "constrained repeated measures (cRM) with heterogeneous variance";

***analysis 6- conditional longitudinal analysis (fix arm term to be 0)***;

**proc** **mixed** data=mixeddata method=reml order=data cl=wald;

class time subj;

model y=time2 trt*time2/ddfm=kenwardroger solution alpha=**0.05**;

repeated time/subject=subj type=un group=trt rcorr;

**run**;
